# Nickel biopathways in tropical nickel hyperaccumulating trees from Sabah (Malaysia)

**DOI:** 10.1038/srep41861

**Published:** 2017-02-16

**Authors:** Antony van der Ent, Damien L. Callahan, Barry N. Noller, Jolanta Mesjasz-Przybylowicz, Wojciech J. Przybylowicz, Alban Barnabas, Hugh H. Harris

**Affiliations:** 1 Centre for Mined Land Rehabilitation, Sustainable Minerals Institute, The University of Queensland, Queensland, Australia; 2 Université de Lorraine–INRA, Laboratoire Sols et Environnement, UMR 1120, France; 3 Deakin University, Geelong, Australia. School of Life and Environmental Sciences, Centre for Chemistry and Biotechnology (Burwood Campus), Victoria, Australia; 4 Materials Research Department, iThemba LABS, National Research Foundation, Somerset West, South Africa; 5 AGH University of Science and Technology, Faculty of Physics & Applied Computer Science, Krakow, Poland; 6 Department of Chemistry, The University of Adelaide, South Australia, Australia

## Abstract

The extraordinary level of accumulation of nickel (Ni) in hyperaccumulator plants is a consequence of specific metal sequestering and transport mechanisms, and knowledge of these processes is critical for advancing an understanding of transition element metabolic regulation in these plants. The Ni biopathways were elucidated in three plant species, *Phyllanthus balgooyi, Phyllanthus securinegioides* (Phyllanthaceae) and *Rinorea bengalensis* (Violaceae), that occur in Sabah (Malaysia) on the Island of Borneo. This study showed that Ni is mainly concentrated in the phloem in roots and stems (up to 16.9% Ni in phloem sap in *Phyllanthus balgooyi*) in all three species. However, the species differ in their leaves – in *P. balgooyi* the highest Ni concentration is in the phloem, but in *P. securinegioides* and *R. bengalensis* in the epidermis and in the spongy mesophyll (*R. bengalensis)*. The chemical speciation of Ni^2+^ does not substantially differ between the species nor between the plant tissues and transport fluids, and is unambiguously associated with citrate. This study combines ion microbeam (PIXE and RBS) and metabolomics techniques (GC-MS, LC-MS) with synchrotron methods (XAS) to overcome the drawbacks of the individual techniques to quantitatively determine Ni distribution and Ni^2+^ chemical speciation in hyperaccumulator plants.

Hyperaccumulators are rare plants that accumulate trace elements to extraordinarily high concentrations in their living aboveground biomass^1^. As of 2016 approximately 450 Ni hyperaccumulators have been documented^2^, mostly in Cuba (130)^3^, Southern Europe and Minor Asia (80–90)^4^,^5^, New Caledonia (65)^6^ and Malaysia (24)^7^. The extreme levels of accumulation of trace elements in hyperaccumulator plants are a consequence of specific metal sequestering and transport mechanisms, and knowledge of these processes is critical in understanding the hyperaccumulation phenomenon. Hyperaccumulator plants are therefore an attractive model for understanding transition element metabolic regulation in plants, because of the extreme expression of this phenotype in such plants. Hyperaccumulator plants also hold much promise for potential utilization in phytomining/agromining. This emerging technology is an environmentally sustainable ‘green’ technology that can extract metals such as Ni from soils with significantly reduced environmental impact compared to conventional mining operations^8^,^9^. In a phytomining/agromining operation, hyperaccumulator plants (‘metal crops’) are grown on sub-economic ores or waste tailings followed by harvesting and incineration of the biomass to generate a commercial high-grade metal bio-ore^10^,^11^,^12^. Nickel phytomining is envisaged to become transformative in the rehabilitation of tropical laterite mining operations as part of the rehabilitation strategies^13^.

Globally the greatest potential for Ni phytomining lies in Indonesia, the Philippines and New Caledonia where extensive ultramafic soils and mining operations exist^13^,^14^. This region has numerous woody hyperaccumulator species that are potentially suitable as ‘metal crops’. Three plant species with particularly high potential are *Phyllanthus balgooyi* Petra Hoffm. & A. J. M. Baker, *Phyllanthus securinegioides* Merr. (Phyllanthaceae) and *Rinorea bengalensis* (Wall.) Kuntze (Violaceae) from Borneo Island^7^,^15^. *Phyllanthus* spp. are scandent shrubs attaining up to 9 m in height, whereas *R. bengalensis* is a tree that grows up to 25 m in height. *Phyllanthus balgooyi* (Phyllanthaceae) was only recently discovered and described^16^ from the Philippines and Malaysian Borneo. This species is highly unusual because its stem exudes a green sap that contains up to 16.9% Ni^17^,^18^. Such plant metal concentrations are only rivalled by the New Caledonian tree *Pycnandra acuminata* (Sapotaceae) which has a latex that can contain up to 25.7% Ni^1^. *Phyllanthus securinegioides* is also a strong hyperaccumulator, its leaves can contain up to 2.3% Ni and has a green phloem, but does not produce copious amounts of Ni-rich exudate as *P. balgooyi*^18^. Finally, *R. bengalensis* also has a green phloem in the trunk which contains up to 5% Ni, whereas the leaves can contain 2.7% Ni^17^.

Studies on the spatial elemental distribution in Ni hyperaccumulator plants have largely focussed on a very limited number of mainly small herbaceous species from the families Asteraceae and Brassicaceae (in the genera *Alyssum, Berkheya, Noccaea, Senecio*)^19^,^20^,^21^,^22^,^23^,^24^,^25^,^26^,^27^,^28^,^29^,^30^ with the exception of *Hybanthus floribundus* (Violaceae), which is a small shrub^31^,^32^ and the tree *Phyllanthus balgooyi*^18^. Leaves were the most often analysed organs^19^,^21^,^22^,^23^,^24^,^25^,^28^,^33^,^34^, while stems^18^,^32^,^35^,^36^,^37^ and roots^21^,^24^,^30^,^36^,^38^,^39^ were seldom measured. In most hyperaccumulator plants Ni is distributed in all tissues but the highest amount is preferentially accumulated in the foliar epidermal cells^19^,^27^,^32^,^34^,^35^,^36^,^37^,^40^. The leaf epidermal vacuoles are the main storage sites of Ni in *Alyssum lesbiacum, A. bertolonii, Noccaea (Thlaspi) goesingense*^19^, *H. floribundus*^31^, *Alyssum murale*^34^ and *Senecio coronatus*^41^. An exception to this rule is *Berkheya coddii* where Ni is accumulated mainly in the mesophyll and in the leaf veins/vascular bundles of the leaf^25^,^28^,^29^,^42^, while the concentrations in the epidermis are lower than the average value for the whole leaf^43^.

In an earlier nuclear microprobe study the highly distinctive spatial distribution of Ni in *P. balgooyi* was reported with extreme levels of accumulation in the vascular bundles of the leaves and phloem tissues in petioles and stems^18^. We expect this type of Ni distribution to be shared with *P. securinegioides* and *R. bengalensis,* on account of the Ni-rich green phloem tissues in all three species.

A combination of Ni^2+^ ligand complexation and compartmentalization at the tissue and cellular-level is involved with tolerance mechanisms in Ni hyperaccumulator plants^22^,^44^. Ligand complexation reduces Ni^2+^ free ion activity and hence cellular-level toxicity^45^. Relevant potential Ni^2+^ ligands include O-donors (carboxylic acids), S-donors (metallothioneins and phytochelatins), and N-donors (histidine, nicotianamine)^46^. In most hyperaccumulator plants Ni^2+^ ligand complexation has been univocally associated with carboxylic acids like citrate and malate^44^,^47^,^48^. Elucidating Ni^2+^ chemical speciation in hyperaccumulator plants has used chromatography methods (GC-MS, LC-MS) and synchrotron X-ray absorption spectroscopy (XAS) methods^44^,^48^. Identification of Ni^2+^ ligand complexes in plant tissues with chromatographic methods is limited by the need to physically destroy and extract compounds from plant tissue samples, potentially leading to alteration of Ni^2+^ coordination complexes in the extract. In addition, identification of Ni^2+^ complexes by electrospray ionisation LC-MS is problematic as complexes can be formed during ionisation, which may not reflect the *in situ* form of Ni^2+^. Synchrotron XAS has the advantage of being able to directly determine Ni^2+^ speciation *in situ* in intact frozen-hydrated plant tissues. However, XAS alone is not able to accurately distinguish between different carboxylic acid Ni^2+^ complexes that are implicated in hyperaccumulation because these complexes provide very little variation in structure to the EXAFS spectra^22^. It is also not possible to determine quantitatively mixed Ni^2+^ complex environments with XAS. Therefore, there is a need to combine quantitative molecular techniques such as GC/LC-MS with the unique ability of XAS to determine *in situ* chemical Ni^2+^ speciation.

This study combines nuclear microprobe (micro-Particle-Induced X-Ray Emission – micro-PIXE and micro-proton backscattering spectrometry – micro-BS) and metabolomics techniques (GC-MS, LC-MS) with synchrotron methods (XAS) to overcome the drawbacks of the individual techniques to quantitatively determine spatial distribution and Ni^2+^ chemical speciation in leaves, stems and roots of hyperaccumulating plants. As such we aim to reveal elemental distribution and Ni^2+^ chemical speciation in the tropical woody hyperaccumulator plants *P. balgooyi, P. securinegioides* and *R. bengalensis*. Specifically, we intend to clarify whether these species have similar patterns of Ni storage and whether Ni^2+^ chemical speciation differs between the species and between plant organs and transport liquids.

## Results

### Ecology of the hyperaccumulating plants

**Figure 1:**
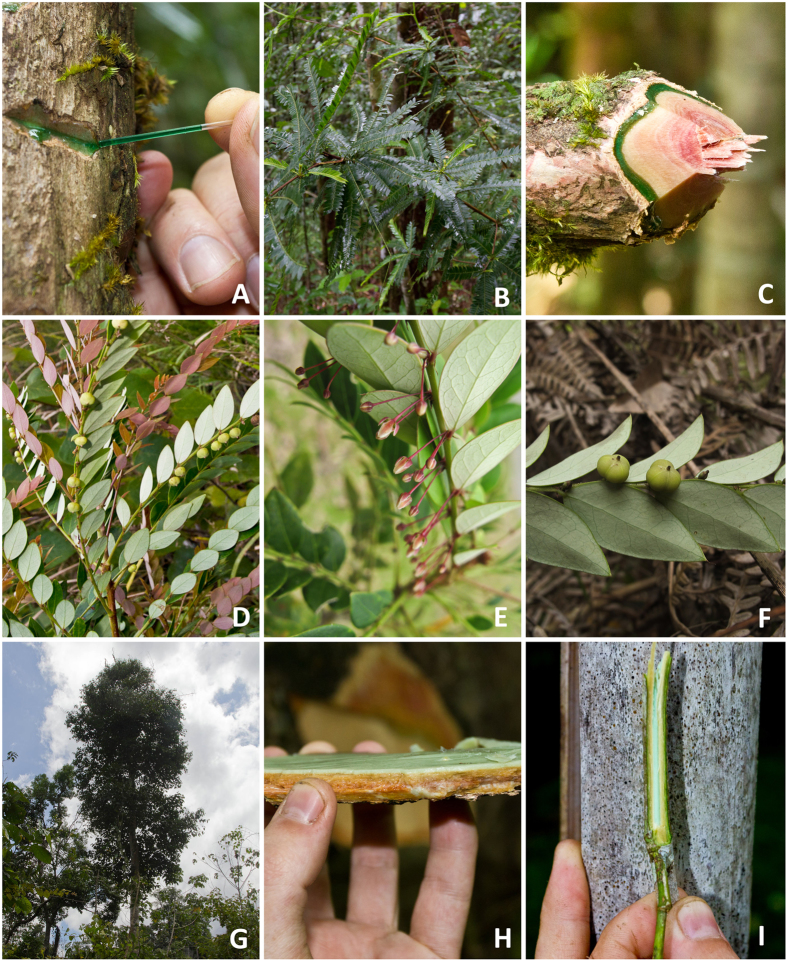
*Phyllanthus balgooyi*: exuding phloem sap (**A)**, branch with leaves (**B**), cut branch with phloem tissue (**C**); *Phyllanthus securinegioides*: branch with leaves and seed capsules (**D**), plant with inflorescences (**E**), seed capsules (**F**); *Rinorea bengalensis*: mature tree 22 m high (**G**), excised bark with phloem tissue (**H**), pith of twig (**I**).

All three species are native to per-humid equatorial rainforest. Figure 1 shows *P. balgooyi, P. securinegioides* and *R. bengalensis* in the native habitat in Sabah (Malaysia) on the Island of Borneo. The soils on which they grow are derived from serpentinised ultramafic bedrock. Concentrations of Ni, Co and Mn are strongly enriched during soil formation. The mean soil pH is rather similar in the rhizosphere from all three species and circum-neutral (pH 6.4–7.1). Although total Ca and Mg concentrations vary, the exchangeable concentrations are similar between the rhizospheres. The soils are K-deficient with mean exchangeable K^+^ ranging from 57–120 μg g^−1^. The mean total Ni concentrations are high (2480–3110 μg g^−1^), but potentially plant available concentrations are moderate with 88–220 μg g^−1^ mean DTPA Ni^2+^ (Table 1).

### Elemental concentrations and distribution in hyperaccumulator plants

Bulk analysis by ICP-AES showed that in the three species foliar and tissue concentrations of most elements, except Ni, are mostly unremarkable. However, foliar K concentrations are high (mean 860–8740 μg g^−1^) considering the K-deficient nature of the soil (Table 2). In comparison with the soil Ca concentrations (mean 1330–1600 μg g^−1^ exchangeable Ca), foliar Ca-concentrations (mean 4290–8020 μg g^−1^) are strongly enriched, especially in *R. bengalensis* (Table 2). The high Ni concentrations (mean 5140–7630 μg g^−1^) in the branches, compared to the wood, can be attributed to the presence of phloem tissue, which is extremely high in Ni (mean 8940–65410 μg g^−1^). The phloem sap of *P. balgooyi* is one of the most unusual biological liquids, it contains 12.6–16.9% Ni, and is also enriched in Co (560–2340 μg g^−1^) and Zn (1900–3690 μg g^−1^), but low in major cations such as Ca (660–1960 μg g^−1^) and K (520–1530 μg g^−1^) (Table 3).

**Figure 2:**
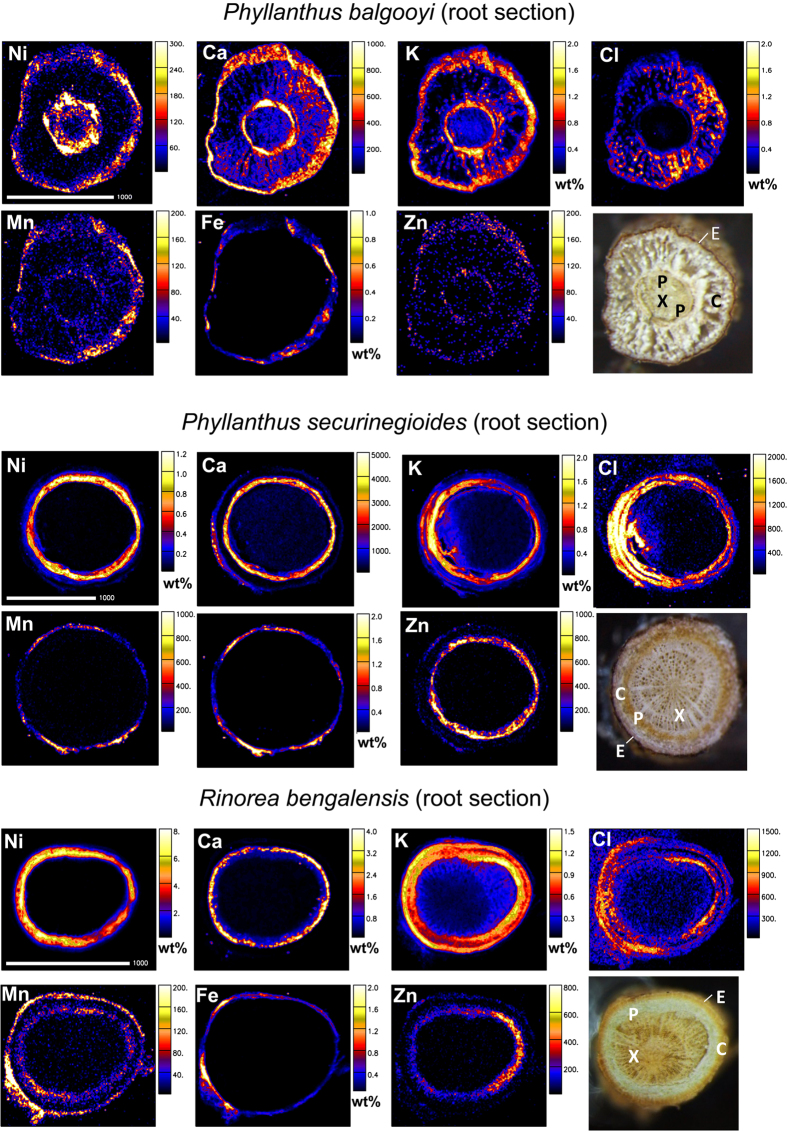
Elemental maps and morphological structure of root cross-sections of *Phyllanthus balgooyi, Phyllanthus securinegioides* and *Rinorea bengalensis.* Concentration scale in wt% dry weight or in μg g^−1^dry weight. X, xylem; P, phloem; C, cortex; E, epidermis. Scale bar – 100 μm.

**Figure 3:**
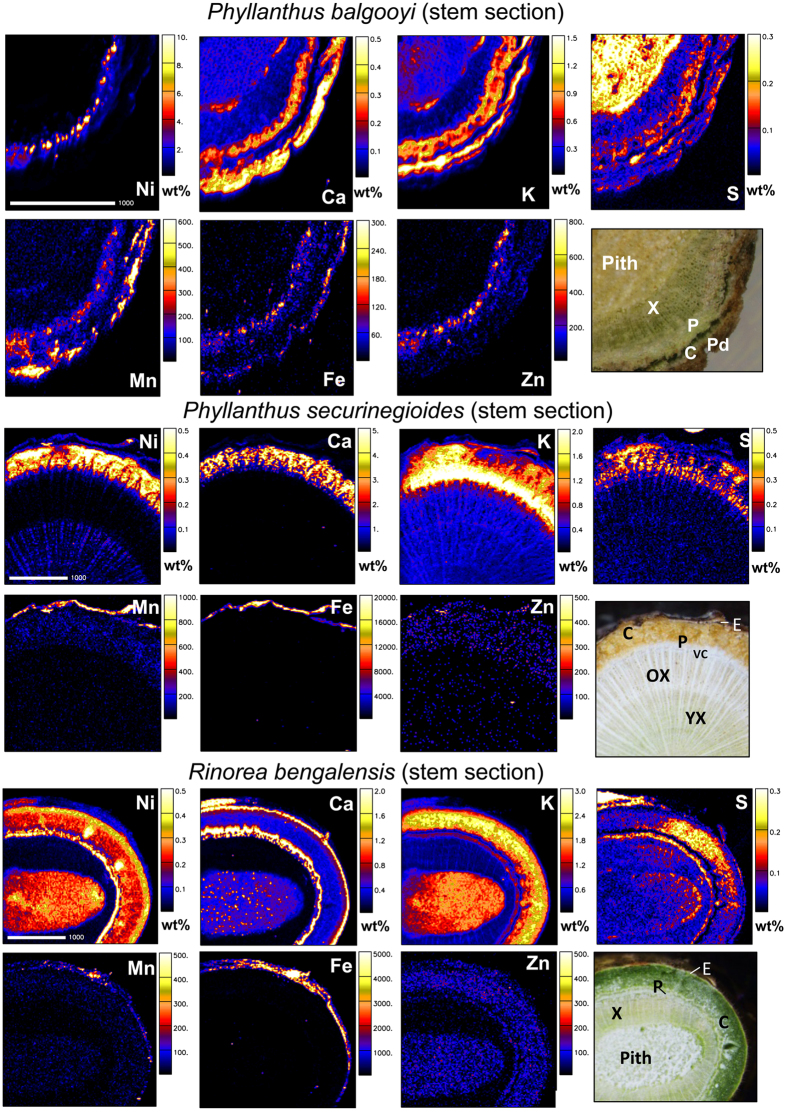
Elemental maps and morphological structure of stem cross-sections of *Phyllanthus balgooyi, Phyllanthus securinegioides* and *Rinorea bengalensis.* Concentration scale in wt% dry weight or in μg g^−1^ dry weight. X, xylem; P, phloem; C, cortex; E, epidermis ; Pd, periderm; VC, vascular cambium; YX, younger xylem; OX, older xylem. Scale bar – 1000 μm.

**Figure 4:**
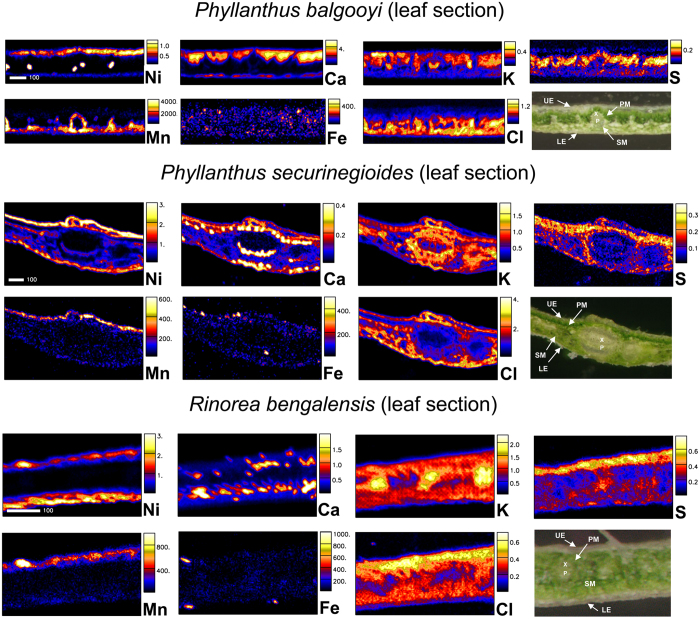
Elemental maps and morphological structure of leaf cross-sections of *Phyllanthus balgooyi, Phyllanthus securinegioides* and *Rinorea bengalensis.* Concentration scale in wt% dry weight (for Ni, Ca, K, S, Cl) and μg g^−1^ dry weight (for Mn, Fe). UE, upper epidermis; LE, lower epidermis; X, xylem; P, phloem; PM, palisade mesophyll; SM, spongy mesophyll. Scale bar – 100 μm.

Micro-PIXE analysis showed that the most important feature of Ni distribution in roots and stems of all three hyperaccumulators is the exceptional enrichment in the phloem (Tables 4 and 5, Figs 2 and 3). However, the species differ in the leaves – in *P. balgooyi* the highest Ni concentration is in phloem, but in *P. securinegioides* in the epidermis. *Rinorea bengalensis* has the highest Ni concentration in the spongy mesophyll but also high enrichment in the epidermis (Table 6, Fig. 4). There are also some other differences. In the roots Ni concentrations in the phloem vary from slightly less than 0.2 wt% in *P. balgooyi* to ca. 0.9 wt% in *P. securinegioides* and 5.6 wt% in *R. bengalensis* (Table 4, Fig. 2). In *P. balgooyi* there is also some enrichment in the epidermis, but the concentrations there are significantly lower than in the phloem (180 μg g^−1^). The lowest amounts of Ni were measured in cortex and xylem. In *P. securinegioides* and *R. bengalensis* the cortex contains more Ni than the epidermis and xylem (Table 4, Fig. 2). Phosphorus, S, K, Ca, and Zn show somewhat similar distribution pattern as Ni, with the highest amounts in the phloem in *P. balgooyi* and *P. securinegioides.* Chlorine is the only element with the highest enrichment in the cortex of *P. balgooyi* but in *P. securinegioides* and *R. bengalensis* the highest concentrations are in the phloem (Table 4, Supplementary Figures 3, 4 and 5). In *R. bengalensis* P and S show markedly different distribution compared to *P. securinegioides* and *P. balgooyi*, forming two enrichment rings in the cortex and xylem, separated by depletion zone in the phloem (Table 4, Supplementary Figures 3, 4 and 5). The highest concentrations of Cl, K, Ca, Cu, Zn, Rb and Sr were found in the phloem. In all three species Si, Ti, Cr, Mn, Fe and Co reach the highest concentrations in the root epidermis.

The elemental distribution in the stems, petioles and leaves of *P. balgooyi* were reported and discussed earlier^18^. The main feature in the stems is the extremely high Ni concentration in the phloem, followed by significant enrichment in the cortex (Table 5, Fig. 3). The distributions of Fe, Co, and Zn mirror that of Ni, but their concentrations are much lower. Although in the stems of *P. securinegioides* and *R. bengalensis* Ni also shows the highest concentrations in the phloem, some important differences were found. The xylem in *P. securinegioides* can be subdivided into younger and older parts, with the younger part significantly enriched in Ni in comparison with the older parts (500 μg g^−1^ and 140 μg g^−1^, respectively) and a very distinct, highly Ni-enriched (up to 2800 μg g^−1^) compressed pith (Table 5). In the stems of *R. bengalensis* the highest Ni concentrations are in the phloem, but equally high values were found in the meristematic cortical cells (Table 5, Fig. 3), of the order of 0.6 wt% in both cases. The remaining parts of cortex also show Ni enrichment (0.3 wt%) as well as the pith (0.28 wt%).

In *P. securinegioides* Ca shows a similar distribution pattern compared to *P. balgooyi* with the highest concentration in the phloem, In *R. bengalensis* it also shows enrichment in the phloem, but the highest concentrations are in the epidermis (Fig. 3, Table 5). Many small “dots” with high Ca amounts are visible in the pith of this species. In *P. securinegioides* K and S are also concentrated in the phloem area, but their distribution pattern is more “diluted” than that of Ni or Ca, and the highest concentration of K is rather in the vascular cambium region. In *R. bengalensis* the distribution of K shows similarities to that of Ni, but the highest concentration of this element is in the cortex.

The distribution pattern of S in *R. bengalensis* is somewhat similar to that of Ni, with the highest concentrations in the phloem and cortex. In *P. securinegioides* P is another element forming an enrichment “ring” in the cambium region (Table 5, Supplementary Figure 6). In *R. bengalensis* this element has a very different distribution pattern, with the highest concentrations in the pith; xylem is the second region of its enrichment, although less pronounced (Table 5, Supplementary Figure 6). Silicon, Ti, Cr, Mn, Fe and Co are only enriched in the epidermal area of stems, both in *P. securinegioides* and *R. bengalensis*. Cl is concentrated in the phloem, but it is not evenly distributed in this region (Supplementary Figure 6). In *P. securinegioides* it forms an enrichment ring in the outer phloem and inner cortex, but in *R. bengalensis* such a ring is visible in the innermost part of phloem.

In comparison with the leaves of *P. balgooyi*, Ni concentrations in the epidermal in *P. securinegioides* and *R. bengalensis* are much higher than in the phloem (Table 6, Fig. 4), although there is some enrichment in the phloem, relative to the adjoining xylem in *P. securinegioides*. In *P. securinegioides* the upper epidermis is notably richer in Ni (up to 4 wt%, on average) than the lower epidermis with the adjoining part of spongy mesophyll (on average 1.9 wt%). In *R. bengalensis* the highest concentrations are in the spongy mesophyll and the adjoining lower epidermis. The upper epidermis has slightly lower concentrations, as well as palisade mesophyll. Among elements showing enrichment in epidermal regions, Mn in *P. securinegioides* is only concentrated in the upper epidermis, while in *R. bengalensis* such enrichment extends to the adjoining palisade mesophyll. Iron enrichment in the epidermal regions is much more “patchy” and less pronounced. Calcium in *P. securinegioides* shows the highest enrichment in the phloem, but in both species it is also concentrated in the mesophyll showing a “grainy” inhomogeneous distribution. The distributions of P and Zn are shown in the Supplementary Figure 7.

### Chemical speciation of Ni^2+^ in hyperaccumulator plants

**Figure 5:**
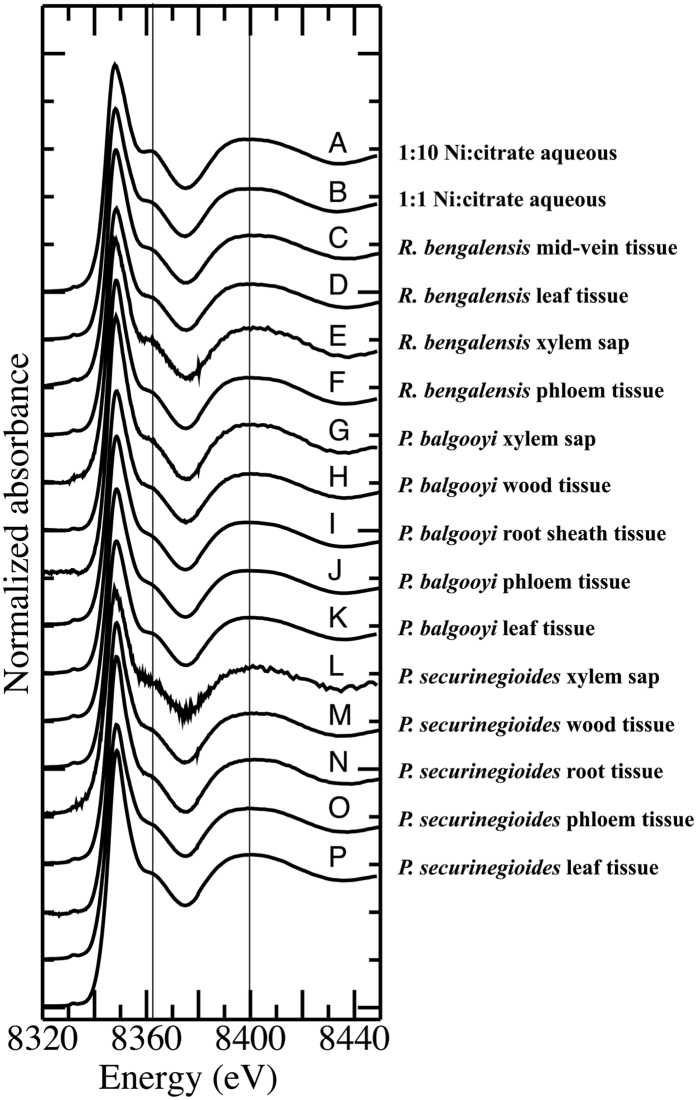
Ni K-edge X-ray absorption near edge spectra for **A** 1:10 Ni:citrate in aqueous solution; **B** 1:1 Ni:citrate in aqueous solution; **C** *R. bengalensis* mid-vein tissue; **D** *R. bengalensis* leaf tissue; **E** *R. bengalensis* xylem; **F** *R. bengalensis* phloem tissue; **G** *P. balgooyi* xylem; **H** *P. balgooyi* wood tissue; **I** *P. balgooyi* root sheath tissue; **J** *P. balgooyi* phloem tissue; **K** *P. balgooyi* leaf tissue; **L** *P. securinegioides* xylem; **M** *P. securinegioides* wood tissue; **N** *P. securinegioides* root tissue; **O** *P. securinegioides* phloem tissue; **P** *P. securinegioides* leaf tissue. Vertical lines are drawn at 8361 and 8400 eV to aid visual comparison of key features in the spectra. The spectra are shifted vertically for clarity.

X-ray Absorption spectroscopy (XAS) was used to investigate the *in situ* Ni speciation in various tissues across the three plant species. The Ni K-edge XANES spectra of the tissues are presented in Fig. 5 (traces **C**-**P**) and visual inspection reveals that the spectra vary little beyond variations in noise level that arise as a result of the varying Ni concentration in the respective tissues. A principal component analysis (PCA) of these fourteen plant spectra suggested that between two and four significant components were present, while visualisation of the PCA eigenvectors (not shown) indicated that the variation was limited to <8350 eV.

**Figure 6:**
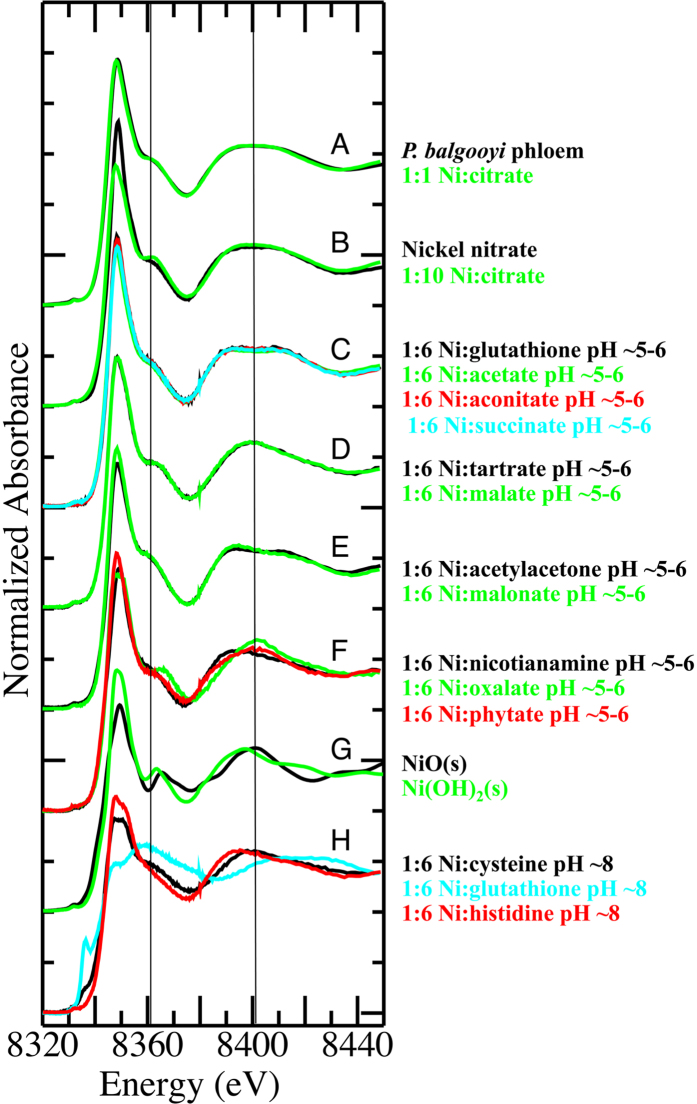
Ni K-edge X-ray absorption near edge spectra for **A** *P. balgooyi* phloem tissue (black trace) and 1:1 Ni:citrate in aqueous solution (green trace); **B** Ni:nitrate in aqueous solution (i.e. [Ni(H_2_O)_6_]^2+^-black trace) and 1:10 Ni:citrate in aqueous solution (green trace); **C**, 1:6 Ni:glutathione in aqueous solution at pH ~5–6 (black trace), 1:6 Ni:acetate in aqueous solution at pH ~5–6 (green trace), 1:6 Ni:aconitate in aqueous solution at pH ~5–6 (red trace), 1:6 Ni:succinate in aqueous solution at pH ~5–6 (blue trace)–all traces essentially identical; **D** 1:6 Ni:tartrate in aqueous solution at pH ~5–6 (black trace), 1:6 Ni:malate in aqueous solution at pH ~5–6 (green trace); **E** 1:6 Ni:acetylacetone in aqueous solution at pH ~5–6 (black trace), 1:6 Ni:malonate in aqueous solution at pH ~5–6 (green trace); **F** 1:6 Ni:nicotianamine in aqueous solution at pH ~5–6 (black trace), 1:6 Ni:oxalate in aqueous solution at pH ~5–6 (green trace), 1:6 Ni:phytate in aqueous solution at pH ~5–6 (red trace); **G** NiO_(s)_ (black trace) and Ni(OH)_2(s)_ (green trace) diluted to 1000 ppm in boron nitride; **H** 1:6 Ni:cysteine in aqueous solution at pH ~8 (black trace), 1:6 Ni:glutathione in aqueous solution at pH ~8 (blue trace), 1:6 Ni:histidine in aqueous solution at pH ~8 (red trace). Vertical lines are drawn at 8361 and 8400 eV to aid visual comparison of key features in the spectra. The spectra are shifted vertically for clarity.

The plant XANES spectra were compared against a number of aqueous solution model complexes of Ni and physiological relevant ligands as well as two insoluble Ni compounds (Fig. 6). Several of the model spectra were easily distinguished from the plant spectra by visual inspection, including complexes with N- or S-donor ligands (Fig. 6 - trace **H**), the solids (trace **G**) as well as the complexes with nicotianamine, oxalate or phytate (trace **F**). The remaining spectra of complexes dominated by carboxylate binding matched the plant-based spectra far more closely, but could be differentiated by features centred at either ~8361 and 8400 eV. The lower energy feature varied in intensity between the different models, being more intense and well resolved in the citrate (1:10 Ni:citrate–trace **B**), tartrate and malonate spectra (trace **D**), but less so in the remaining spectra including the plant-based example in **A**.

Variation in the higher energy feature was more subtle, being either: broad and rounded (1:10 Ni:citrate–trace **B**); narrower and rounded (malate and tartrate in **D**); split and resolved symmetrically (the four traces in **C**); split and resolved asymmetrically (acetylacetonate and malonate in trace **E**); or broad and unresolved (the plant and 1:1 Ni:citrate model in trace **A**).

Meanwhile, the spectrum of hexaquo Ni^2+^ showed intermediate intensity of the peak at 8361 eV and a broad unresolved feature centred on 8400 eV. While this region was very similar to the plant-based spectra, the white line peak (~8349 eV) intensity for the hexaquo complex was significantly greater than in either the plant or carboxylate model spectra, as well as appearing at slightly higher energy as previously reported by others^44^.

In summary, the plant XANES spectra were well matched by the spectrum of the 1:1 solution of Ni(II) and citrate at pH 5.5, and could be distinguished from the spectra of all other model complex spectra by a combination of features at the white line, 8361 and ~8400 eV. The XANES spectra for pH adjusted and unadjusted Ni:citrate were consistent.

We did attempt a least-squares fitting of the plant spectra with a selection of the model complex XANES spectra but abandoned this approach for several reasons. As noted above, a principal component analysis was not conclusive regarding the number of model compounds that should be included in the linear regression and fit results generated with different numbers of components varied markedly. In addition, and perhaps most importantly, the spectrum of the 1:1 Ni:citrate solution was recorded at a different beamline (the AS XAS beamline; as the ANBF was permanently decommissioned shortly after the spectra of the plants and other models were recorded) than the spectra of the plant tissues. Because the spectral resolution of the two beamlines was not necessarily identical (also potentially affecting energy calibration) the spectrum of the 1:1 citrate model could not be rigorously included in the statistical analyses, despite being apparently the best match by eye.

We also compared the extended X-ray absorption structure (EXAFS) data of the plant tissues (Supplementary Figure 1 - all recorded at ANBF) with the EXAFS data from a selection of the most closely matched model spectra (Supplementary Figure 2 - recorded at the AS) according to the XANES results. We note that the EXAFS data will be less sensitive to differences in the spectral resolution of the two beamlines. Visual inspection of the k-space data to 10 Å^−1^ in Supplementary Figure 1 reveals that the EXAFS for these samples are essentially identical with minor variations noted between 3–4 and 5–6 Å^−1^, particularly for the leaf samples (black traces **F** and **I**). More significant variations were noted in these ranges for the model complexes EXAFS data shown in Supplementary Figure 2 with those EXAFS and variations similar to those previously reported by others^22^,^44^. Only the EXAFS of the malonate complex was clearly distinguishable from that of the plant samples (due to a strong oscillation in the EXAFS at ~3.5 Å^−1^) but the EXAFS of the plants was closest to that of the 1:1 citrate model (asymmetrical oscillation between 3–4 Å^−1^ and a weak “shoulder” on the low-k side of the oscillation between 5–6 Å^−1^). Again, attempts to fit the plant sample EXAFS with a linear combination of the model complex EXAFS was abandoned because variation in the number of fitted models dramatically altered the fit results.

Taken together, the XANES and EXAFS data of the plants indicates that the predominant species present in the tissues is a 1:1 Ni complex with citrate and the remainder of the coordination sphere consisting of water ligands. The minor variations in the spectra in the leaf tissues can be explained by the presence of a 1:2 complex of Ni with citrate, producing a slightly more intense peak in the XANES at 8361 eV (Fig. 5–traces **P** and **K**) and, somewhat less clearly, a variation in the EXAFS between 5–6 Å^−1^. It is certainly feasible that the relative proportion of these two species may vary between plant species and between particular tissues in those species. We believe that the more complicated mixtures of ligands identified by other groups in other Ni hyperaccumulators, including malate complexes and others, may be as a result of the fact that they did not include the 1:1 Ni:citrate complex spectrum in their fitting, and the evidence for the presence of these other complexes based purely on XAS data is weak.

**Figure 7:**
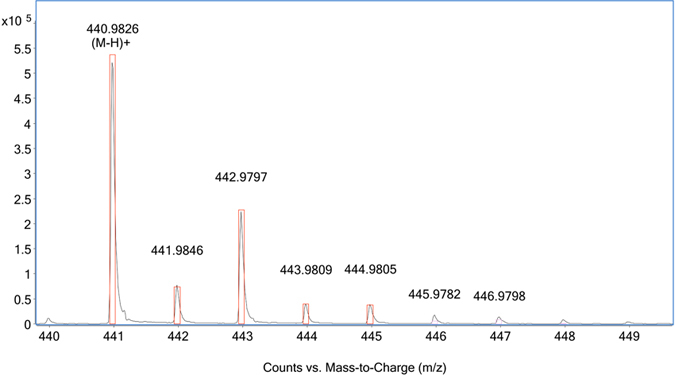
Mass spectrum of Ni-citrate [M.L_2_-H]^+^ from *P. balgooyi* phloem sap (black trace) and theoretical isotope pattern for Ni-citrate (red boxes).

Gas-chromatography (GC-MS) was used for untargeted metabolite profiling in the freeze-dried plant tissues and phloem sap samples. Relative response ratios (RRR’s) were calculated using the metabolite peak area divided by the internal standard area and sample mass. This resulted in a list of 95 compounds, of which mass spectral libraries positively identified 60 compounds (Table 7), however the method cannot detect any Ni-complexes. GC-MS was also used to quantify the six most abundant metabolites determined in the metabolomic profiles; these were malate, citrate, fructose, glucose, sucrose and catechin in the *P. balgooyi* phloem sap (Table 8). The sugars are of interest, as ref. 48 reported the formation of Ni:citrate:sucrose complexes. Ion chromatography (IC) was then used to quantify anions in the *P. balgooyi* phloem sap and as such compile a mass balance of ligands and anions available to complex Ni^2+^ (Table 9). The pH of the thawed phloem sap was pH 6.54, which aligned well with spot tests taken in the field on fresh sap (~pH 6.0–6.5). The use of LC-MS enabled the identification Ni^2+^ containing complexes. A large number of ions containing the isotope pattern of Ni^2+^ were present in the chromatograms of the extracts of the plant species. Figure 7 shows the mass spectrum of Ni-citrate [M.L_2_-H]^+^ from *P. balgooyi* phloem sap (black trace) and theoretical isotope pattern for Ni-citrate (red boxes). The most prominent Ni^2+^ containing ion detected in all samples was Ni^2+^ citrate [M.L_2_-H]^+^.

The mass balance presented in Table 9 shows that the mean anion concentration is 0.46 wt% versus a mean of 76.4 wt% citrate to balance a mean of 15.6 wt% Ni. Taking into account relevant ionic charges, the quantities of citrate together with the relatively minor concentrations of cations are sufficient to complex and ionically balance virtually all of Ni^2+^ present in the phloem sap of *P. balgooyi*.

## Discussion

All three species have a highly distinctive tissue Ni distribution patterns with extreme levels of accumulation in the phloem of the root and stem. The phloem tissues in the main stem of all three species are green due to the exceptionally high concentration of Ni^2+^ ions (Fig. 1) and appear to act as a ‘sink’ with Ni concentrations reaching up to 2.1% in *R. bengalensis* and up to 16.9 wt%, in the phloem sap from *P. balgooyi* (Table 2). The difference occurs in leaves – in *P. balgooyi* the highest Ni concentration is in the phloem, but in *P. securinegioides* and *R. bengalensis* in the epidermis and in the spongy mesophyll (*R. bengalensis)*. Nickel accumulation in the leaf epidermal cells of Ni hyperaccumulators appears to be a global trait, with the exception of *B. coddii* and now *P. balgooyi*. Although high accumulation in the vascular bundles is not an anomaly among herbaceous hyperaccumulating plants, most previous investigations concentrated on leaf epidermal tissue as important storage site and did not pay much attention to Ni transport and concentration in the vascular bundles. High concentrations of Ni in the phloem tissue in roots and stems are not only a feature of woody tropical hyperaccumulator plants; significant Ni enrichment in the phloem was reported from the stem of *Senecio coronatus*^26^, *Alyssum murale*^21^ and in bark/phloem boundary in *A. lesbiacum* and *A. bertolonii*^19^. High Ni concentrations were also found in the phloem tissues of *Senecio coronatus* roots^30^, vascular cylinders of young *Alyssum murale* roots^21^,^24^ and root vascular tissues of *Berkheya coddii*^39^. High Ni concentrations in vascular bundles were also measured in the leaves of *B. coddii*^29^,^43^, *Stackhousia tryonii*^37^ and *Alyssum murale*^21^,^22^. Significant enrichment of Ni in phloem exudates from *Noccaea caerulescens* leaves were recently reported^49^. These reports and results of our present investigations suggest that high concentration of Ni in phloem could be a common feature in Ni hyperaccumulator plants and this requires more attention in further investigations.

Preferential accumulation of Ni in vascular tracts suggests that Ni is present in water-soluble form. The chemical speciation of Ni^2+^ has been implicated to play a significant role in tissue and cellular-level detoxification, but the observation that Ni^2+^ complexes are water-soluble (as evidenced from the phloem sap) ostensibly suggest that Ni^2+^ is present in a metabolically active form. Although it has been suggested that Ni^2+^ may be complexed with S and N-donors^50^, the tissue concentrations of S are orders of magnitude lower than Ni in the three species studied, and unlikely to be important. Nevertheless, in *Alyssum* spp. SO_2_^−4^ was posited as counter ion for Ni^2+^ in vacuoles, rather than S-containing organic ligands^19^,^34^.

The results from the XAS analyses here show that Ni^2+^ is complexed by carboxylic acids (predominantly citrate) in all three species and in all different tissues and transport liquids analysed. The Ni^2+^ chemical speciation differs very little between the species and between the tissues, except for small changes in the relative abundance of Ni:citrate complexes of 1:1 and 1:2 stoichiometry. This aligns with titrimetric determinations of Ni:citrate complexes^51^. It, therefore, appears that the chemical speciation of Ni^2+^ does not change as it is transported from the roots to the leaves.

Due to the earlier alluded difficulties of accurately determining the stoichiometry of the carboxylic acids involved using synchrotron XAS alone, we have coupled the analyses with chromatographic methods. The only Ni^2+^ complex that could be identified using LC-MS analysis was the Ni^2+^ citrate complex [M.L_2_-H]^+^, whereas GC-MS and HPLC-UV analysis quantified the concentrations of carboxylic acids. The phloem sap fluid is ‘biologically relevant’ as it derived directly from sieve elements and hence exists in that form within intact cells. As has been established in other tropical hyperaccumulator plants with Ni-rich phloem tissues, Ni^2+^ is complexed by citrate (though note that in *Pycnandra acuminata* this is in laticifer exudates). Citrate is the most abundant carboxylic acid in plants and is produced through the Krebs-cycle and the metabolic cost of citric acid as a ligating agent for Ni^2+^ is presumably low. Even though this oxygen-donor has a low association constant with Ni^2+^ (logK 5.4), S and N-donors with higher stability constants, such as histidine (logK 8.7) or nicotianamine (logK 16.1), are simply not economic for plants to be produced at sufficient quantities, and are not detected in quantities in our experiments. Furthermore, at the acidic pH of plant cell vacuoles (pH < 5.5), carboxylic acids are better ligands for Ni^2+^ than amino acids^52^.

Other studies have found evidence for Ni^2+^ complexation with histidine, phytate and phosphate using XAS (for example^50^,^53^), however the inability of this method to quantify the stoichiometry between Ni^2+^ and ligands means that interpretation of this data is difficult. The role of citrate in Ni hyperaccumulation has long been recognised. Studies by ref. 47, 48, 54 and 55 showed that in 12 different New Caledonian hyperaccumulator species Ni^2+^ was almost exclusively ligated with citrate (and minor malate). The remarkable green latex of *Pycnandra acuminata* latex has been studied numerous times which revealed that 37–99% of Ni^2+^ is ligated by citrate^56^,^57^,^58^.

This study has shown how accelerator/ion beam, synchrotron and metabolomics techniques can be combined to iteratively strengthen the understanding of Ni distribution and chemical speciation in hyperaccumulating plant tissues. Ni^2+^ ligation with citrate is a common characteristic of the studied hyperaccumulator plants, with extreme accumulation in the phloem bundles in these tropical woody species. As such Ni is concentrated from the foliar phloem bundles into phloem tissue in branches and the main trunk. The levels of concentration of the Ni biopathway through the plants are remarkable, in *P. balgooyi* with a mean of 56 μg g^−1^ Ni in the xylem increasing to a mean of 14.9% Ni in the phloem sap. As opposed to xylem, movement of ions through the phloem is bi-directional and could hence facilitate Ni^2+^ re-distribution in the hyperaccumulator plant to emerging shoots and leaves (as shown by ref. 49 for *Noccaea caerulescens*). It is unknown how the extreme concentrations of Ni in the phloem vessels affect the osmotic pressure of the sieve elements and therefore pressure flow involved with phloem loading and unloading of sugars.

The weak nature of the Ni^2+^ citrate complex means that in a mixed ligand environment with N and S-donors Ni^2+^ would cause enzyme function inhibition and hence toxicity. Therefore, physical separation of the Ni^2+^ citrate complex from metabolically sensitive cell organs is still necessary. The preferential accumulation in the vacuoles of foliar epidermal cells and associated apoplastic space appears to be typical for most hyperaccumulator plants. In these locations Ni^2+^ is separated from photosynthesis at the tissue-level and basic metabolic processes at the cell-level.

Hyperaccumulation essentially consists of two discrete stages, abnormal uptake behaviour in the root with enhanced translocation, followed by effective tissue and cell-level sequestration. Although the Ni^2+^ complex environment throughout the studied hyperaccumulating plants is rather simple (Ni^2+^ citrate), there are still major knowledge gaps in understanding the biopathway leading from Ni^2+^ uptake in the rhizosphere. The results of this study have shown that once Ni^2+^ enters the xylem the bulk chemical speciation of Ni^2+^ does not change substantially, however, that raises the question how Ni^2+^ is trafficked through root apoplastic and symplastic pathways with such exceptional selectivity. For example, the phloem tissue of *R. bengalensis* contains on average 17 790 μg g^−1^ Ni versus on average 30 μg g^−1^ Co (or 1:593), while soil concentrations are in the order of 1:10. The differences in the association constants for the citrate complexes of Fe, Ni, Co do not permit selective uptake of Ni^2+ ^46. Even if citric acid is involved with ligating Ni^2+^ in root exudates, only cell-membrane transport proteins based on S-donors have sufficient selectivity and binding strength to traffic Ni^2+^ across the pathways in the root.

The viability of Ni phytomining depends on the combination of biomass production per time unit and Ni uptake-accumulation of the target plant^59^. The maximum Ni concentration in the biomass is important, but the speed at which Ni^2+^ is taken up and transferred to the growing plant and incorporated into the biomass is also an important factor. A detailed understanding of this process may allow for manipulations in a phytomining operation to increase Ni^2+^ uptake efficiency, for example by application of specific soil amendments.

## Materials and Methods

### Collection and bulk analysis of plants

Plant samples (roots, wood, phloem tissue, leaves and phloem and xylem sap) and rhizosphere soil samples of *P. balgooyi, P. securinegioides* and *R. bengalensis* were collected in the native rainforest habitat in Borneo (near Kinabalu Park in Sabah, Malaysia). Bark, phloem and wood samples were taken from the main trunks of all three species by excising these parts with a sharp stainless steel surgical knife and separating them. Phloem sap was collected with glass capillary tubes (200 μm inner diameter 50 mm long), whereas xylem sap was collected with a handheld vacuum pump from excised branches. Individual tissue samples intended for synchrotron XAS (X-ray Absorption Spectroscopy) analysis were subsequently extracted with a razor blade, placed in polycarbonate cuvettes, covered with Kapton tape and rapidly frozen by immersion in liquid N_2_ (−196 °C) in the field. No other processing of samples for XAS, such as homogenization, was undertaken and the samples were maintained at cryogenic temperatures between collection in the field and data collection at the synchrotron. Tissue samples intended for nuclear microprobe (micro-PIXE) analysis were similarly excised, but immediately shock-frozen using a metal mirror technique in which the samples were pressed between a block of Cu-metal cooled by liquid N_2_ and a second Cu-metal block attached to a Teflon holder. This ensured extremely fast freezing of the plant tissue samples to prevent cellular damage by ice crystal formation. The samples were then wrapped in Al-foil, and transported in a cryogenic container (kept at −196 °C).

Parallel samples (~300 mg) of all the types described above were freeze-dried (−50 °C for 24 h) and digested using 4 mL HNO_3_ (70%) and 1 mL H_2_O_2_ (30%) in a microwave oven and diluted to 30 mL with ultrapure water (Millipore 18.2 MΩ·cm at 25 °C). Air-dried rhizosphere soil samples were similarly analysed, but digested with 6 mL HNO_3_ (70%) and 3 mL HCl (30%). These samples then were analysed on ICP-AES (Inductively Coupled Plasma Atomic Emission Spectroscopy) for macro-element and trace elements.

### Nuclear microprobe (PIXE) elemental mapping

The frozen tissue samples were freeze-dried in a Leica EM CFD Cryosorption Freeze Dryer (Leica Microsystems AG, Austria). The freeze-drying process followed a long, 208 h programmed cycle to prevent shrinkage of the tissues. Freeze-dried plant tissues were then hand cut with a steel razor blade and mounted on specimen holders covered with 0.5% Formvar film and lightly coated with carbon to prevent charging. Several sections from three replicas of each species and organs were obtained. The most representative were analyzed using the nuclear microprobe. Microanalyses were performed using the nuclear microprobe at the Materials Research Department, iThemba LABS, South Africa. The facility and methodology of measurements of biological materials have been reported elsewhere in detail^60^,^61^. To summarize: a proton beam of 3 MeV energy, provided by a 6 MV single-ended Van de Graaff accelerator, was focused to a 3 × 3 μm^2^ spot and raster scanned over the areas of interest, using square or rectangular scan patterns with a variable sizes (from 400 μm × 400 μm to 2.7 mm × 2.7 mm) and variable number of pixels (up to 128 × 128). The proton current was restricted to 100–150 pA to minimize specimen beam damage. Particle-induced X-ray emission (PIXE) and proton backscattering spectrometry (BS) were used simultaneously. PIXE spectra were registered with a Si(Li) detector (30 mm^2^ active area and 8.5 μm Be window) with an additional 125 μm Be layer as an external absorber. The effective energy resolution of the PIXE system (for the Mn Kα line) was 160 eV, measured for individual spectra. The detector was positioned at a takeoff angle of 135° and a working distance of 24 mm. The X-ray energy range was set between 1 and 40 keV. BS spectra were recorded with an annular Si surface barrier detector (100 μm thick) positioned at an average angle of 176°. Data were acquired in the event-by-event mode. The normalization of results was performed using the integrated beam charge, collected simultaneously from a Faraday cup located behind the specimen and from the insulated specimen holder. In total 25 scans were performed (8 leaves, 9 stems and 8 roots). The results reported in figures and corresponding tables are from representative scans done for roots, stems and leaves, respectively. More experimental details can be found elsewhere^18^.

### Synchrotron (XAS) Ni chemical speciation analysis

Nickel K-edge XAS spectra of the plant tissue samples and standards were recorded in fluorescence mode at either the Australian National Beamline Facility (ANBF), Beamline 20B (bend magnet) at the Photon Factory (2.5 GeV) KEK, Tsukuba, Japan, or at the XAS beamline at the Australian Synchrotron (AS). At the ANBF, mono-chromation was achieved using a water-cooled channel-cut Si(111) crystal monochromator with a 1 mm beam size in the horizontal and 0.5 mm in the vertical direction, and harmonic rejection was achieved by detuning to 50% intensity. At the AS the X-ray beam was mono-chromated by diffraction from a pair of Si(111) crystals and harmonic rejection was achieved with the use of a Rh-coated mirror.

At both beamlines fluorescence data were recorded using a Canberra-Eurisys 36-element Ge-detector (the same detector was used at both beamlines) positioned at 90ᵒ to the incident beam, with the sample positioned at 45ᵒ to the incident beam. The X-ray beam energy was calibrated simultaneously with data collection using a Ni metal foil recorded in transmission downstream of the sample, where the first peak of the first derivative was assumed to 8331.6 eV.

At the ANBF the data were collected with 10 eV steps over the pre-edge region from 8120–8320 eV and 0.25 eV steps over the edge region from 8320–8380 eV followed by increments in k-space of 0.05 Å^−1^ until k was equal to either 7 (for the standards) or 12 Å^−1^ (for the plant samples). Similar parameters were used at the AS, except that the pre-edge point spacing was 6 eV and the k-space steps were 0.035 Å^−1^ to k = 12 Å^−1^.

All samples and standards were analysed at between 5 and 15 K using a closed-cycle He cryostat and contained in either polycarbonate or poly-lactic acid cuvettes covered with Kapton tape. A total of 17 aqueous Ni^2+^ standards were prepared by adding ligands in calculated molar excess (1:6) to Ni^2+^ to ensure the formation of Ni-ligand complexes (excepting a standard of Ni:nitrate dissolved in water where no additional ligands were added and a standard prepared with a 1:1 ratio of Ni:citrate which was only analysed at the AS). The solutions were diluted to 5 mM [Ni^2+^] before analysis. The pH of the standards was not adjusted, except for the Ni:citrate standards which were prepared at unadjusted and pH adjusted with NaOH to pH 5.5, and with the exception of histidine and glutathione which were adjusted to pH 8 to allow for full de-protonation and hence coordination via N and S donor atoms respectively). Glycerol was then added (final concentration 33 v/v%) to each standard before flash freezing in liquid nitrogen.

EXAFS data to k = 12 Å^−1^ was subsequently recorded at the AS for 6 standards (citrate (for both 1:1 and 10:1 citrate:Ni ratios), malate, malonate, tartate, and hexaquo), with a ligand:Ni ratio of 10:1 and with pH adjusted to 5.5 using NaOH. A comparison of the XANES spectra with the ANBF data equivalents showed that they were identical within the noise level in each case.

### Metabolite profiling with GC-MS and LC-MS/HPLC-UV

GC-MS was used for untargeted metabolite profiling in the plant tissues and phloem sap. Freeze-dried samples were accurately weighed into 2 mL cryo-mill tubes containing ceramic beads then extracted with 1 mL of 50% methanol using the Preselys cryo-mill with the following settings: temperature 5 °C, speed 3800 rpm, time 3 × 30 s. Tubes were centrifuged and supernatant transferred to 2 mL vials for analysis. The GC-MS system used comprised an AS 3000 auto-sampler, a Trace gas chromatograph Ultra and a DSQ quadrupole MS with an EI source (ThermoElectron Corporation, Austin, USA). The MS was tuned according to the manufacturer’s recommended procedure using tris-(perfluorobutyl)-amine (FC43). Derivatives were separated on a 30 m VF-5MS column (0.25 mm, 10 m Integra guard column; 0.25 μm film thickness; Varian Inc., Victoria, Australia) with a helium carrier gas (1 mL min^−1^). For trimethylsilyl (TMS) derivatives the following oven temperature program was used: start temperature at 70 °C followed by a 1 °C min^−1^ oven temperature ramp to 76 °C, then a 7 °C min^−1^ ramp to 325 °C and finally held at 325 °C for 10 min. For tert-butyldimethylsilylation (TBS) derivatives the following oven program was used: start temperature at 100 °C and hold for 1 min then 1 °C min^−1^ ramp to 106 °C followed by a 7 °C min^−1^ ramp to 325 °C and finally held for 10 min at 325 °C. The following MS conditions were used: injection temperature 23 °C, MS transfer line 280 °C and ion source 250 °C. Mass spectra were recorded at 2 scan/s, with a scanning range of 70–600 amu. For quantitative analysis of the key high abundant metabolites (malate, citrate, fructose, glucose, sucrose and catechin) standard curves were produces using authentic standards.

Citrate quantification was carried out using HPLC with UV detection at 210 nm (Dionex Ultimate 3000). Extracts were injected (10 μL) onto a Phenomonex Acclaim OA 150 × 3mm μm column. Samples were eluted using an isocratic mobile phase at 0.4 mL min^−1^ containing 100 mM Na_2_SO_4_, pH 2.65 (adjusted with methanesulfonic acid). External calibration standards were used to quantify the total citrate concentrations.

LC-MS was used to search for potential Ni-complexes in the aqueous extracts. An Agilent 6520 electrospray ionisation quadrupole time of flight (ESI-QTOF) high-resolution mass spectrometer was coupled to and Agilent 1200 series liquid chromatography (LC) system. A 2.1 × 100 mm, 1.8 μm C18 Zorbax Elipse plus (Agilent), reversed phase column was used for chromatography. The LC parameters were as follows: column temperature 30 °C, flow rate 0.4 mL min^−1^, with gradient elution using mobile phase A 0.1% formic acid in water, mobile phase B 0.1% formic acid in acetonitrile. The initial mobile phase composition was 1% B which was changed linearly to 100% B over 10 minutes with a 2 minute hold at 100% B then re-equilibration for 5 min at 2% B, giving a total run time of 17 mins. The ESI source settings were: gas temperature 300 °C, gas flow rate 10 L min^−1^, nebulizer pressure 45 psi, capillary voltage 4000 V, fragmentor 150 V. Instrument was tuned in extended dynamic range mode and spectra collected between 70–1700 m/z at 2 scans/second. Samples were analysed in both positive and negative ionization mode.

Ion chromatography (IC) was used to determine the concentrations of anions in the *P. balgooyi* phloem sap. The freeze-dried phloem sap was reconstituted in ultrapure water, filtered with a 0.45 μm syringe filter and anions analysed using a Dionex IC instrument. The pH of the *P. balgooyi* phloem sap was determined using a micro-tip pH instrument (TPS, Ag/AgCl with ceramic frit junction).

### Data processing and statistics

Quantitative results for the PIXE were obtained by a standardless method using GeoPIXE II software package^62^,^63^,^64^. The error estimates were extracted from the error matrix generated in the fit, and the minimum detection limits (MDL) were calculated using the Currie equation^65^. Quantitative elemental mapping was performed using Dynamic Analysis method^64^,^66^,^67^. This method generates elemental images, which are (i) overlap-resolved, (ii) with subtracted background and (iii) quantitative, *i.e.* in μg g^−1^ dry weight units. Maps were complemented by data extracted from arbitrarily selected micro-areas corresponding to separated tissues within roots, stems and leaves. PIXE spectra from these micro-areas were used to obtain average elemental concentrations using a full nonlinear deconvolution procedure of fitting PIXE spectra^62^,^63^, with matrix corrections based on thickness and matrix composition obtained from the corresponding BS spectra, fitted with a RUMP simulation package^68^ with non-Rutherford cross-sections for C, O, N. Elemental concentrations from these areas are also reported in μg g^−1^ dry weight.

Data reduction and analysis of the XAS analysis, including calibration, averaging and background subtraction of all spectra and principal component analysis (PCA), target transformation and linear regression analyses of XANES spectra, were performed using the EXAFSPAK software package (G. N. George, Stanford Synchrotron Radiation Lightsource, Menlo Park, CA, USA). All individual detector channels from repeat scans were first averaged using the EXAFSPAK subroutine “average” and the averaged data was then energy shifted according to the Ni foil calibration described above. Background subtraction was then carried out on the averaged data using default values for the Ni K edge in the EXAFSPAK subroutine “backsub”. PCA, target transformation and linear combination fits of XANES spectra were performed over the Ni K-edge region (8320–8450 eV). Linear combination fits did not provide consistent reliable results as noted above and as such these results are not reported herein. Small changes in the presence or absence of minor component models in the fits lead to significant changes in fit results.

The GC-MS data were processed using AMDIS (Automated Mass spectral Deconvolution and Identification System; NIST, Gaithersburg, USA) deconvolution was carried out on a chromatogram for each species to identify as many known and unknown chromatographic peaks as possible. The eluting derivatives were identified using the National Institute of Standards and Technology (NIST 2008) commercial mass spectra library, and the public domain mass spectra library the Max-Planck-Institute for Plant Physiology, Golm^69^. Processing methods were created using the Xcalibur program (ThermoFinnigan), which determined the areas under each peak, found following deconvolution in AMDIS. The processing method used the mass spectral pattern and RT relative to n-alkanes to enable reliable identification in all samples. In the processing method setup, metabolites, which were not identified through library searching, were labelled with their corresponding RT values. In the LC-MS data ions containing Ni^2+^ were identified using the unique theoretical isotope pattern. Peak areas for each Ni^2+^ -containing ion were determined using MassHunter quant (Agilent).

## Additional Information

**How to cite this article**: van der Ent, A. *et al*. Nickel biopathways in tropical nickel hyperaccumulating trees from Sabah (Malaysia). *Sci. Rep.* **7**, 41861; doi: 10.1038/srep41861 (2017).

**Publisher's note:** Springer Nature remains neutral with regard to jurisdictional claims in published maps and institutional affiliations.

## Supplementary Material

Supplementary Information

## Figures and Tables

**Table 1:** Elemental concentrations in the rhizosphere and in the bedrock in the natural habitat of *Phyllanthus balgooyi, Phyllanthus securinegioides* and *Rinorea bengalensis* values as ranges and means in μg g^−1^ and *wt% dry weight).

Species	n	Rhizosphere soil chemistry							
		pH	Mg (total)*	Mg (exch.)	P (total)	K (total)	K (exch.)	Ca (total)	Ca (exch.)
*Phyllanthus balgooyi*	3	6.4–6.5 [6.4]	6.93–9.92 [8.58]	1040–3520 [1970]	82–180 [140]	153–179 [163]	32–100 [57]	180–1500 [640]	340–3320 [1590]
*Phyllanthus securinegioides*	9	5.6–7.3 [6.6]	3.33–14.71 [7.56]	510–6120 [2732]	44–590 [200]	36–328 [102]	17–200 [67]	90–7720 [3750]	450–4440 [1600]
*Rinorea bengalensis*	6	6.6–7.6 [7.1]	0.20–5.40 [2.45]	710–4790 [2190]	45–540 [200]	32–144 [91]	21–310 [120]	63–9240 [2820]	360–3460 [1330]
**Species**	**n**	**Cr (total)**	**Mn (total)**	Fe (total)*	**Co (total)**	**Co (DTPA)**	**Ni (total)**	**Ni (DTPA)**	**Ni (Sr(NO**_**3**_)_**2**_
*Phyllanthus balgooyi*	3	420–440 [430]	1432–2330 [1770]	5.42–7.71 [6.35]	170–560 [360]	16–68 [40]	1620–3960 [2480]	180–290 [220]	4.2–48 [20]
*Phyllanthus securinegioides*	9	1240–6050 [3080]	1427–6460 [3010]	4.03–18.55 [9.83]	240–710 [430]	5–24 [15]	1080–5450 [2570]	21–200 [88]	2.1–15 [7.4]
*Rinorea bengalensis*	6	1920–10980 [4710]	2200–7330 [4060]	7.92–39.60 [16.12]	210–690 [350]	4–24 [11]	2160–4410 [3110]	21–440 [150]	3.4–17 [9.2]
**Species**	**n**	**Bedrock chemistry**							
		Na*	Mg*	Al*	Si*	**P**	S*	K*	Ca*
*Phyllanthus balgooyi*	3	0.02–0.15 [0.08]	38–46 [42]	0.4–4.5 [2.5]	16–20 [18]	11–72 [42]	0.04–0.1 [0.07]	0.01–0.01 [0.01]	0.8–2.4 [1.6]
*Phyllanthus securinegioides*	3	0.01–0.13 [0.06]	7.0–46 [29]	0.2–2.8 [1.3]	13–20 [17]	30–52 [38]	0.05–0.1 [0.07]	0.01–0.03 [0.02]	0.2–1.7 [0.7]
*Rinorea bengalensis*	2	0.12–0.20 [0.16]	9.0–18 [14]	0.7–2.4 [1.6]	2–21 [12]	38–76 [57]	0.06–0.09 [0.07]	0.01–0.03 [0.02]	1.8–2.6 [2.2]
**Species**	**n**	**Cr**	**Mn**	Fe*	**Co**	**Ni**	**Cu**	**Zn**	**Mo**
*Phyllanthus balgooyi*	3	750–2830 [1790]	1170–5770 [3470]	6.0–10 [8.2]	9.2–20 [15]	1110–1390 [1250]	0.7–35 [18]	54–120 [85]	1.0–1.5 [1.2]
*Phyllanthus securinegioides*	3	850–2230 [1730]	890–2540 [1630]	1.9–8.5 [5.9]	9.0–11 [10]	1030–1610 [1380]	8–14 [10]	49–59 [55]	1.2–1.6 [1.4]
*Rinorea bengalensis*	2	1661–2630 [2140]	930–1300 [1120]	4.3–4.6 [4.5]	6.8–8.8 [7.8]	970–1220 [1090]	43–50 [47]	46–68 [57]	0.9–1.3 [1.1]

The digests and extracts were analysed with ICP-AES. Abbreviations: *‘ICP-AES’* is Inductively Coupled Plasma Atomic Emission Spectroscopy), ‘*Total’* is extractable with HCl/HNO_3_ digest, ‘*DTPA’* is extractable with Diethylenetriamine pentaacetic acid solution, ‘*Exch*.’ is extractable with silver thiorea solution, and *‘Sr(NO*_*3*_)_*2*_’ is extractable with strontium nitrate solution.

**Table 2:** Bulk elemental concentrations in plant tissues (leaves, twigs, bark, wood) and fluids (xylem and phloem) in *Phyllanthus balgooyi, Phyllanthus securinegioides* and *Rinorea bengalensis* (values as ranges and means in μg g^−1^ dry weight).

Species	n	Ni	Mg	Al	P	S	K	Ca	Co	Mn	Zn
**Leaves**											
*Phyllanthus balgooyi*	7	1070–8290 [4680]	1060–4980 [3080]	2.0–46 [31]	31–740 [370]	120–1380 [860]	72–364 [860]	230–8680 [4290]	6.0–65 [31]	63–480 [240]	14–150 [88]
*Phyllanthus securinegioides*	14	10750–25060 [16530]	1060–5500 [3410]	14–69 [31]	430–1690 [740]	1200–2690 [1570]	3100–13380 [5630]	3010–12160 [5790]	12–75 [30]	95–460 [190]	45–250 [92]
*Rinorea bengalensis*	12	2390–8470 [5470]	1470–5130 [3060]	10–70 [26]	420–1190 [790]	1040–2700 [1640]	2160–27990 [8740]	4210–20480 [8020]	10–32 [19]	90–910 [370]	33–280 [95]
**Branches**											
*Phyllanthus balgooyi*	3	2550–8610 [5270]	270–500 [390]	10–5 [29]	140–200 [180]	320–700 [520]	1190–2320 [1780]	630–3740 [2070]	11–46 [26]	19–190 [89]	35–94 [64]
*Phyllanthus securinegioides*	6	5110–12310 [7630]	330–5280 [1740]	8.0–55 [29]	250–1450 [780]	320–1330 [760]	4920–13250 [8710]	1180–25860 [8290]	5.0–36 [20]	38–150 [94]	46–100 [74]
*Rinorea bengalensis*	4	1810–8510 [5140]	1080–6040 [3010]	5.0–69 [28]	280–1170 [720]	680–2350 [1510]	1990–11430 [6520]	2040–77430 [34410]	9.0–20 [13]	16–110 [57]	27–250 [120]
**Wood**											
*Phyllanthus balgooyi*	3	250–880 [560]	90–250 [150]	14–31 [20]	62–78 [67]	210–280 [240]	330–490 [420]	430–490 [460]	2.0–11 [5.7]	5.0–47 [20]	8.0–14 [11]
*Phyllanthus securinegioides*	7	440–1170 [860]	85–250 [150]	2.8–23 [14]	25–790 [360]	50–510 [310]	80–2720 [1580]	125–1300 [760]	0.8–15 [5.0]	5.9–18 [11]	4.8–14 [10]
*Rinorea bengalensis*	5	340–1920 [880]	290–650 [470]	6.0–26 [13]	110–360 [210]	250–1100 [440]	510–2720 1470]	1720–5760 [3360]	2.0–14 [4.8]	6.0–21 [13]	7.0–100 [35]
**Bark**											
*Phyllanthus balgooyi*	3	210–370 [310]	170–420 [310]	23–93 [55]	14–25 [21]	71–110 [90]	23–54 [42]	50–120 [90]	2.8–5.2 [4.0]	22–45 [37]	5.9–11 [8.8]
*Phyllanthus securinegioides*	5	3770–6820 [5860]	400–2160 [870]	14–58 [38]	230–510 [390]	630–1130 [830]	1860–6240 [4420]	1960–23400 [10850]	4.4–14 [9.2]	33–89 [68]	74–83 [78]
*Rinorea bengalensis*	2	3300–4630 [3970]	570–650 [610]	49–70 [59]	220–390 [310]	500–770 [640]	2890–3120 [3000]	28260–35530 [31890]	4.9–5.2 [5.1]	25–48 [36]	227–250 [240]
**Roots**											
*Phyllanthus balgooyi*	1	4780	300	14	95	680	610	410	120	7	120
*Phyllanthus securinegioides*	1	1270	380	91	40	81	53	130	26	16	24
*Rinorea bengalensis*	—	n.a.	n.a.	n.a.	n.a.	n.a.	n.a.	n.a.	n.a.	n.a.	n.a.

The digests and extracts were analysed with ICP-AES.

**Table 3:** Bulk elemental concentrations in transport tissue and liquids (xylem and phloem) in *Phyllanthus balgooyi, Phyllanthus securinegioides* and *Rinorea bengalensis* (values as ranges and means in μg g^−1^ dry weight).

Species	n	Ni	Mg	Al	P	S	K	Ca	Co	Mn	Zn
**Xylem exudate**											
*Phyllanthus balgooyi*	**2**	35–76 [56]	4.0–11 [7.5]	<LOD	3.0–7.0 [5.0]	7.0–10.0 [8.5]	44–52 [48]	10–30 [20]	0.01–1.0 [0.5]	1.0–1.0 [1.0]	0.01–1.0 [0.5]
*Phyllanthus securinegioides*	**1**	13	2.0	<LOD	5.0	2.0	53	<LOD	<LOD	<LOD	<LOD
*Rinorea bengalensis*	**2**	6.0–19 [12.5]	3.0–7.0 [5.0]	<LOD	4.0–5.0 [4.5]	3.0–6.0 [4.5]	28–58 [43]	10–10 [10]	<LOD	<LOD	<LOD
**Phloem sap**											
*Phyllanthus balgooyi*	**4**	125910–168510 [148730]	490–820 [680]	6.0–30 [19]	23–190 [130]	470–920 [730]	520–1530 [980]	660–1960 [1450]	560–2340 [1550]	320–480 [390]	1900–3690 [2580]
**Phloem tissue**											
*Phyllanthus balgooyi*	**4**	45010–79340 [65410]	600–1160 [890]	12–18 [15]	140–240 [220]	1310–2150 [1850]	2700–4770 [3320]	2920–4410 [3570]	190–1170 [575]	160–280 [200]	410–1930 [960]
*Phyllanthus securinegioides*	**5**	5990–10800 [8940]	1060–1360 [1230]	16–29 [25]	170–340 [260]	950–2740 [1760]	3670–8420 [5650]	12860–51510 [38820]	12–24 [19]	62–130 [87]	67–190 [97]
*Rinorea bengalensis*	**7**	13630–22600 [17790]	650–3020 [1380]	12–27 [20]	260–760 [470]	450–4630 [2960]	4770–8670 [7270]	3670–37210 [22570]	4–58 [30]	29–290 [290]	210–770 [510]

The digests were analysed with ICP-AES.

**Table 4:** Elemental concentrations (micro-PIXE, μg g^−1^ dry weight) in the morphological structures of the roots of *Phyllanthus balgooyi, Phyllanthus securinegioides* and *Rinorea bengalensis*.

	Ni	Si	P	S	Cl	K	Ca	Mn	Fe	Co	Cu	Zn
*Phyllanthus balgooyi*												
Whole area	120 (4)	2600 (180)	100 (16)	1310 (40)	5040 (12)	6870 (50)	410 (23)	29 (3)	710 (15)	7 (3)	3.1 (0.5)	10.4 (0.7)
Epidermis	180 (14)	35010 (14)	<113	1120 (50)	320 (25)	2380 (50)	860 (27)	63 (9)	3540 (50)	17 (12)	<15	15 (7)
Cortex	34 (3)	450 (3)	90 (20)	840 (20)	7780 (20)	7950 (70)	380 (26)	9 (2)	163 (8)	<2	<2.1	10 (1)
Primary phloem	1880 (30)	<385	330 (60)	1320 (40)	1550 (30)	10480 (40)	900 (24)	29 (4)	<10	9 (5)	<22	23 (6)
Primary xylem	25 (5)	<160	230 (36)	610 (40)	890 (30)	4470 (30)	150 (20)	<4	<4	<3	<6	<8
*Phyllanthus securinegioides*												
Whole area	2080 (43)	8170 (470)	240 (32)	2420 (100)	570 (40)	4400 (80)	820 (20)	44 (2)	1230 (20)	6 (4)	6 (2)	113 (2)
Epidermis	670 (13)	34600 (1500)	75 (24)	1320 (20)	360 (16)	1590 (40)	460 (10)	230 (8)	8330 (110)	50 (13)	<11	18 (4)
Cortex	2910 (50)	1350 (180)	330 (30)	3520 (130)	750 (30)	3300 (50)	250 (10)	24 (3)	440 (7)	<3	8 (3)	47 (5)
Phloem	9250 (120)	190 (40)	450 (50)	5220 (240)	1360 (60)	11960 (120)	3250 (40)	7 (2)	47 (3)	7 (6)	10 (5)	570 (12)
Xylem	340 (9)	390 (40)	300 (30)	1120 (50)	230 (12)	2700 (20)	280 (9)	2.8 (0.7)	14.7 (0.5)	<0.6	2.1 (0.5)	17.7 (0.7)
*Rinorea bengalensis*												
Whole area	14700 (150)	6310 (650)	130 (23)	1580 (90)	370 (20)	5570 (60)	5460 (50)	25 (2)	920 (15)	<5	25 (7)	63 (2)
Epidermis	270 (8)	11860 (800)	73 (14)	480 (12)	220 (13)	780 (17)	590 (10)	70 (3)	3170 (50)	13 (5)	<3	29 (2)
Cortex	10060 (140)	<128	220 (30)	2020 (110)	480 (20)	9460 (130)	5530 (100)	5 (3)	132 (5)	<5	21 (8)	23 (4)
Phloem	56400 (400)	<110	93 (25)	1450 (80)	690 (13)	10560 (80)	8410 (60)	33 (3)	67 (2)	<11	78 (21)	250 (20)
Xylem	910 (14)	260 (14)	220 (20)	420 (25)	140 (8)	2660 (20)	1150 (8)	2.6 (0.6)	6.4 (0.5)	<0.8	3.6 (0.6)	13.5 (0.8)

The results were obtained from PIXE spectra extracted from regions representing different morphological structures, and analysed using GeoPIXE software. Errors of analysis (±1 σ uncertainty) are shown in brackets.

**Table 5:** Elemental concentrations (micro-PIXE, μg g^−1^ dry weight) in the morphological structures of the stems of *Phyllanthus balgooyi, Phyllanthus securinegioides* and *Rinorea bengalensis*.

	Ni	Si	P	S	Cl	K	Ca	Mn	Fe	Co	Cu	Zn
***Phyllanthus balgooyi***												
Whole area	6860 (80)	<45	315 (16)	1510 (50)	2970 (60)	5280 (50)	1920 (23)	115 (3)	13 (1)	22 (3)	22 (4)	56 (2)
Outer periderm	2010 (30)	170 (55)	310 (23)	940 (40)	970 (20)	3740 (30)	4100 (26)	331 (3)	28 (1)	12 (2)	17 (2)	47 (3)
Inner periderm	4150 (60)	280 (60)	260 (26)	860 (25)	6500 (90)	14680 (120)	2040 (50)	119 (4)	<3	27 (3)	22 (4)	66 (4)
Cortex	12800 (170)	<125	260 (15)	910 (40)	5700 (100)	7570 (80)	1400 (20)	101 (5)	14 (3)	40 (6)	34 (8)	106 (4)
Phloem	86300 (400)	n.d.	<300	1060 (50)	4200 (90)	6930 (50)	2680 (24)	202 (11)	43 (12)	265 (26)	<200	405 (27)
Xylem	1450 (22)	260 (40)	350 (40)	760 (40)	680 (16)	2350 (24)	760 (9)	31.2 (0.9)	7 (1)	3 (1)	11 (1)	12 (1)
Pith	840 (19)	<93	370 (30)	3000 (80)	3710 (30)	4570 (60)	1330 (22)	40 (1)	1.8 (0.8)	2 (1)	10 (1)	13 (1)
***Phyllanthus securinegioides***												
Whole area	1040 (12)	1840 (200)	365 (20)	830 (50)	730 (11)	8550 (100)	6660 (70)	48 (3)	335 (8)	5 (3)	<2	14.1 (0.7)
Epidermis	700 (24)	42600 (1800)	130 (60)	640 (20)	160 (12)	2360 (60)	2080 (40)	460 (26)	10910 (180)	47 (20)	<12	26 (6)
Cortex	960 (20)	1060 (200)	270 (40)	770 (50)	2560 (40)	6630 (30)	2130 (30)	109 (6)	1460 (23)	18 (5)	<9	24 (6)
Secondary phloem	4150 (60)	1090 (220)	90 (70)	2340 (140)	2280 (50)	14540 (210)	35100 (320)	67(4)	15 (4)	<9	<8	25 (3)
Vascular cambium	530 (23)	920 (140)	1460 (100)	1420 (70)	825 (40)	28800 (100)	2390 (60)	39 (3)	11 (3)	<9	8 (3)	37 (3)
Older xylem	140 (5)	750 (100)	440 (60)	336 (18)	106 (10)	5240 (20)	500 (10)	10.6 (0.9)	11 (0.8)	<1.1	3.3 (0.9)	7.8 (0.9)
Younger xylem	500 (12)	800 (120)	300 (50)	325 (30)	49 (7)	3760 (14)	530 (14)	14.1 (0.9)	21 (1)	<1.2	3.2 (0.9)	5 (1)
Compressed pith	2830 (80)	830 (210)	370 (110)	770 (50)	58 (26)	8810 (80)	4050 (50)	62 (8)	<9	<11	<18	48 (11)
***Rinorea bengalensis***												
Whole area	1780 (11)	1680 (300)	1550 (170)	760 (40)	120 (20)	11150 (180)	5720 (90)	15 (1)	170 (7)	4 (4)	7 (2)	40 (2)
Epidermis/phellem	1430 (70)	3530 (330)	300 (40)	640 (40)	76 (12)	5030 (160)	12180 (120)	122 (9)	2340 (100)	14 (11)	<8.	21 (4)
Cortex	3070 (40)	1960 (380)	370 (80)	1180 (80)	165 (25)	22420 (190)	4980 (80)	12 (1)	19 (2)	<9	6 (3)	69 (2)
Meristematic cortical cells	6540 (80)	1420 (230)	290 (50)	1220 (50)	160 (25)	22530 (180)	5550 (70)	9 (3)	<11	<15	<27	76 (10)
Phloem	6430 (40)	1400 (340)	1600 (200)	1230 (60)	330 (18)	11550 (230)	8300 (120)	10 (3)	38 (5)	<9	<17	70 (9)
Xylem	367 (15)	1130 (230)	2000 (190)	650 (30)	150 (16)	4440 (60)	690 (16)	4.4 (0.5)	8.8 (0.7)	<1	18 (1)	10.4 (1)
Pith	2820 (40)	1810 (400)	4210 (420)	810 (40)	126 (19)	17500 (220)	6230 (80)	14 (1)	8 (1)	<4	21 (3)	62 (3)

The results were obtained from PIXE spectra extracted from regions representing different morphological structures, and analysed using GeoPIXE software. Errors of analysis (±1 σ uncertainty) are shown in brackets.

**Table 6:** Elemental concentrations (micro-PIXE, μg g^−1^ dry weight) in the morphological structures of the leaves of *Phyllanthus balgooyi, Phyllanthus securinegioides* and *Rinorea bengalensis*.

	Ni	P	S	Cl	K	Ca	Mn	Fe
*Phyllanthus balgooyi*								
Whole area	3050 (50)	520 (40)	1100 (40)	6430 (60)	2160 (30)	16330 (130)	830 (15)	38 (3)
Upper epidermis	9000 (130)	<130	410 (40)	2130 (130)	1710 (30)	33930 (270)	150 (10)	32 (8)
Palisade mesophyll	160 (13)	590 (60)	850 (70)	10540 (90)	1630 (30)	3050 (40)	1910 (40)	27 (11)
Xylem	200 (40)	<244	300 (90)	4580 (130)	2250 (80)	1960 (80)	390 (50)	43 (28)
Phloem	29400 (350)	680 (80)	1140 (70)	9730 (120)	2190 (40)	3100 (50)	2700 (60)	<120
Spongy mesophyll	1210 (45)	790 (60)	1900 (40)	5250 (30)	3300 (50)	37700 (210)	230 (9)	51 (10)
Lower epidermis	1930 (60)	490 (60)	960 (50)	7700 (60)	1340 (30)	17460 (150)	480 (17)	29 (10)
*Phyllanthus securinegioides*								
Whole area	9490 (150)	500 (30)	1120 (70)	16070 (200)	9000 (140)	2300 (60)	41 (5)	16 (3)
Upper epidermis	39500 (600)	<470	1630 (90)	16840 (170)	3340 (70)	1780 (40)	480 (28)	85 (18)
Palisade mesophyll	1700 (50)	1450 (100)	2240 (80)	7700 (90)	9470 (70)	2910 (60)	21 (8)	35 (9)
Xylem	650 (19)	750 (40)	450 (30)	6240 (60)	9400 (120)	330 (40)	5 (3)	<3
Phloem	8200 (150)	420 (80)	640 (70)	11510 (140)	14400 (100)	9500 (80)	<11	<12
Spongy mesophyll	4820 (110)	<360	630 (70)	14460 (260)	10040 (130)	970 (50)	<22	<23
Lower epidermis	19010 (200)	490 (100)	1130 (70)	27800 (200)	6200 (70)	3840 (50)	<32	<36
*Rinorea bengalensis*								
Whole area	7100 (100)	1030 (60)	2980 (100)	3740 (70)	12030 (90)	4870 (50)	110 (4)	36 (3)
Upper epidermis	11800 (140)	460 (40)	5110 (90)	4770 (40)	7420 (60)	33530 (40)	400 (14)	20 (5)
Palisade mesophyll	9210 (110)	860 (60)	5690 (120)	6130 (50)	13300 (100)	3970 (40)	300 (9)	23 (10)
Xylem	1100 (30)	980 (110)	1710 (90)	3590 (60)	21090 (110)	2060 (60)	15 (7)	19 (8)
Phloem	1170 (40)	1430 (110)	1800 (100)	3740 (60)	24050 (150)	2830 (110)	<14	19 (8)
Spongy mesophyll	17800 (190)	1600 (100)	2800 (80)	3850 (43)	13680 (70)	4060 (50)	56 (4)	14 (5)
Lower epidermis	13700 (150)	240 (24)	1930 (40)	2310 (43)	4480 (35)	1090 (30)	40 (3)	59 (4)

The results were obtained from PIXE spectra extracted from regions representing different morphological structures, and analysed using GeoPIXE software. Errors of analysis (±1 σ uncertainty) are shown in brackets.

**Table 7:** Major metabolites and identified Ni-complexes in leaf and phloem extracts of *P. balgooyi, P. securinegioides* and *R. bengalensis*.

Metabolites	Plant tissue and phloem sap samples			
	*Phyllanthus balgooyi* (phloem tissue)	*Phyllanthus balgooyi* (phloem sap)	*Rinorea bengalensis* (phloem tissue)	*Phyllanthus securinegioides* (phloem tissue)
	Mean (n = 3)	Mean (n = 3)	Mean (n = 3)	Mean (n = 3)
***Relative Response Ratios** ( **RRR’s***)				
Pyruvic acid	0.0372 ± 0.0108	0.0417 ± 0.0139	0.0154 ± 0.0041	0.0096 ± 0.0049
Lactate	0.0399 ± 0.0093	0.0126 ± 0.0139	0.0615 ± 0.0147	0.0483 ± 0.0201
Glycolic acid	0.0170 ± 0.0017	0.0151 ± 0.0167	0.0096 ± 0.0010	0.0149 ± 0.0002
Alanine 2TMS	0.0070 ± 0.0010	0.0012 ± 0.0015	0.0261 ± 0.0021	0.0098 ± 0.0016
Glycine	0.0008 ± 0.0001	0.0005 ± 0.0006	0.0051 ± 0.0008	0.0010 ± 0.0001
butyro-1,4-lactam 1TMS	0.0130 ± 0.0003	0.0017 ± 0.0035	0.0466 ± 0.0084	0.0174 ± 0.0026
Valine	0.0105 ± 0.0015	0.0037 ± 0.0063	0.0331 ± 0.0038	0.0174 ± 0.0013
Benzoate	0.0615 ± 0.0039	0.0438 ± 0.0537	0.0273 ± 0.0021	0.0456 ± 0.0019
Serine	0.0050 ± 0.0009	0.0003 ± 0.0005	0.0239 ± 0.0028	0.0045 ± 0.0011
Glycerol/Phosphate	0.3294 ± 0.1643	0.0292 ± 0.0698	0.9054 ± 0.0902	0.4143 ± 0.0671
Isoleucine	0.0030 ± 0.0009	0.0000 ± 0.0000	0.0197 ± 0.0020	0.0056 ± 0.0009
Nicotinic acid	0.0266 ± 0.0008	0.0086 ± 0.0109	0.0254 ± 0.0019	0.0125 ± 0.0014
Glycine	0.0015 ± 0.0005	0.0004 ± 0.0004	0.0083 ± 0.0005	0.0018 ± 0.0005
Succinate	0.0115 ± 0.0008	0.0038 ± 0.0045	0.0310 ± 0.0038	0.0309 ± 0.0059
Glyceric acid	0.0042 ± 0.0001	0.0007 ± 0.0008	0.0131 ± 0.0024	0.0037 ± 0.0004
Uracil	0.0297 ± 0.0045	0.0122 ± 0.0166	0.0173 ± 0.0020	0.0211 ± 0.0015
Itaconic acid	0.0247 ± 0.0027	0.0169 ± 0.0143	0.0219 ± 0.0002	0.0156 ± 0.0048
Fumarate	0.0216 ± 0.0021	0.0053 ± 0.0078	0.0490 ± 0.0050	0.0235 ± 0.0044
Serine	0.0132 ± 0.0007	0.0016 ± 0.0023	0.0848 ± 0.0091	0.0132 ± 0.0018
3-cyano-alanine	0.0006 ± 0.0001	0.0005 ± 0.0008	0.0007 ± 0.0000	0.0009 ± 0.0001
Threonine	0.0079 ± 0.0014	0.0011 ± 0.0019	0.0223 ± 0.0020	0.0085 ± 0.0005
2-methyl maleic acid	0.0101 ± 0.0007	0.0072 ± 0.0072	0.0097 ± 0.0010	0.0108 ± 0.0029
Aspartate	0.0052 ± 0.0019	0.0000 ± 0.0005	0.0081 ± 0.0004	0.0011 ± 0.0003
Glutamine	0.0009 ± 0.0001	0.0001 ± 0.0001	0.0058 ± 0.0009	0.0006 ± 0.0002
Malate	0.1190 ± 0.0168	0.0132 ± 0.0130	0.8612 ± 0.0925	0.3969 ± 0.0923
Erythritol	0.0022 ± 0.0009	0.0002 ± 0.0007	0.0101 ± 0.0025	0.0037 ± 0.0007
Aspartate	0.2400 ± 0.0841	0.0063 ± 0.0265	1.1451 ± 0.0441	0.0748 ± 0.0297
Pyroglutamate	0.3796 ± 0.0432	0.0706 ± 0.1181	1.0969 ± 0.0553	0.5092 ± 0.1199
4-amino-butyric acid	0.0477 ± 0.0070	0.0009 ± 0.0009	0.1910 ± 0.0102	0.1033 ± 0.0054
Glutamate	0.0252 ± 0.0057	0.0024 ± 0.0040	0.1892 ± 0.0169	0.0943 ± 0.0217
4-hydroxy-benozic acid	0.2051 ± 0.0177	0.0933 ± 0.1161	0.0787 ± 0.0038	0.1048 ± 0.0056
Xylose	0.0010 ± 0.0001	0.0000 ± 0.0000	0.0194 ± 0.0032	0.0010 ± 0.0002
1,6-anhydroglucose	0.0059 ± 0.0007	0.0036 ± 0.0070	0.0093 ± 0.0006	0.0116 ± 0.0017
Xylitol	0.0095 ± 0.0013	0.0000 ± 0.0000	0.0070 ± 0.0006	0.0094 ± 0.0016
cis-aconitic acid	0.0797 ± 0.0135	0.0202 ± 0.0235	0.0497 ± 0.0017	0.0104 ± 0.0035
Glycerol-3-phosphate	0.0000 ± 0.0000	0.0000 ± 0.0000	0.0007 ± 0.0001	0.0012 ± 0.0001
4-hydroxy-3-methoxybenzoiac acid	0.1297 ± 0.0797	0.0104 ± 0.0214	0.0709 ± 0.0072	0.0609 ± 0.0173
Pinitol	0.0002 ± 0.0000	0.0001 ± 0.0002	0.7277 ± 0.0526	0.0005 ± 0.0001
Myoinositol	0.0004 ± 0.0000	0.0002 ± 0.0002	0.4952 ± 0.0532	0.0011 ± 0.0001
Galactonic acid	0.0025 ± 0.0008	0.0004 ± 0.0003	0.0226 ± 0.0038	0.0494 ± 0.0092
Saccharic acid	0.0370 ± 0.0126	0.0064 ± 0.0062	0.5932 ± 0.0649	0.0030 ± 0.0012
Mucic acid	0.0126 ± 0.0039	0.0035 ± 0.0092	0.4892 ± 0.0547	0.0032 ± 0.0004
Hexadecanoic acid	0.3215 ± 0.0460	0.1719 ± 0.2136	0.1369 ± 0.0052	0.1817 ± 0.0091
Inositol	0.1708 ± 0.0269	0.0052 ± 0.0046	0.7108 ± 0.0829	0.6813 ± 0.0564
3-deoxy-arabino-hexaric acid	0.0497 ± 0.0037	0.0191 ± 0.0271	0.1238 ± 0.0191	0.0103 ± 0.0015
Galactose	0.0013 ± 0.0003	0.0011 ± 0.0007	0.0727 ± 0.0064	0.0315 ± 0.0009
Maltitol	0.0011 ± 0.0005	0.0001 ± 0.0001	0.0019 ± 0.0016	0.0003 ± 0.0000
Fructose-6-phosphate	0.0007 ± 0.0002	0.0000 ± 0.0000	0.0129 ± 0.0023	0.0029 ± 0.0005
Glucose-6-phosphate	0.0013 ± 0.0004	0.0000 ± 0.0000	0.0404 ± 0.0083	0.0074 ± 0.0007
Trehalose	0.3398 ± 0.3342	0.0003 ± 0.0042	0.3895 ± 0.0051	0.0443 ± 0.0106
Maltose	0.0067 ± 0.0029	0.0004 ± 0.0006	0.0161 ± 0.0046	0.0338 ± 0.0031
Gentobiose	0.0093 ± 0.0021	0.0001 ± 0.0003	0.0418 ± 0.0076	0.0062 ± 0.0005
Catechin	4.4191 ± 0.8557	0.0029 ± 0.0219	0.0140 ± 0.0047	1.3337 ± 0.3064
Melibiose	0.0015 ± 0.0003	0.0001 ± 0.0001	0.0191 ± 0.0016	0.0056 ± 0.0018
Raffinose	0.1009 ± 0.0170	0.0016 ± 0.0021	0.0435 ± 0.0113	0.0732 ± 0.0108
Citrate*	**40.0191 ± 2.1491**	**6.1564 ± 8.4050**	**13.4089 ± 0.6403**	**2.2423 ± 0.1830**
Fructose	0.3981 ± 0.1534	0.0086 ± 0.0085	24.9293 ± 5.2262	0.4236 ± 0.0355
Glucose	0.8223 ± 0.1927	0.0110 ± 0.0091	46.2812 ± 10.4612	0.7154 ± 0.0456
Sucrose	6.3945 ± 2.4236	0.1041 ± 0.0605	32.8001 ± 0.6488	16.7075 ± 1.9097

Results obtained with GC-MS. Nickel complexes identified by LC-MS in bold and indicated by asterisk.

**Table 8:** Concentrations of citric acid, malic acid, fructose, sucrose, glucose and catechin in leaf and phloem extracts of *P. balgooyi, P. securinegioides* and *R. bengalensis*.

Species	Part	Sample	Malate	Citrate	Fructose	Glucose	Sucrose	Catechin
			**μg g**^**−1**^ **(dry weight)**					
*Phyllanthus balgooyi*	Phloem tissue	1	0.8	121.7	0.4	0.5	13.9	5.9
*Phyllanthus balgooyi*	Phloem tissue	2	0.5	81.6	0.4	0.4	10.4	3.9
*Phyllanthus balgooyi*	Phloem tissue	3	0.5	102.5	1.6	1.1	62.4	3.2
*Phyllanthus balgooyi*	Phloem sap	1	12.8	480.0	2.3	2.7	4.9	n.d.
*Phyllanthus balgooyi*	Phloem sap	2	14.1	285.6	2.1	1.5	6.7	n.d.
*Phyllanthus balgooyi*	Phloem sap	3	14.4	314.8	1.5	0.7	11.9	n.d.
*Phyllanthus securinegioides*	Phloem tissue	1	1.1	12.1	0.5	0.4	40.7	1.7
*Phyllanthus securinegioides*	Phloem tissue	2	1.4	14.0	0.4	0.4	43.1	1.7
*Phyllanthus securinegioides*	Phloem tissue	3	1.2	14.9	0.6	0.5	50.9	1.9
*Rinorea bengalensis*	Phloem tissue	1	3.8	70.9	19.4	16.2	186.0	n.d.
*Rinorea bengalensis*	Phloem tissue	2	3.0	34.4	18.1	13.9	141.5	n.d.
*Rinorea bengalensis*	Phloem tissue	3	3.6	41.7	17.7	15.5	135.2	n.d.

Results obtained with quantitative GC-MS.

**Table 9:** Mass-balance including major cations and anions in the phloem sap of *P. balgooyi*.

Mass Balance *Phyllanthus balgooy*i phloem sap							
Sample	Anions μg g^−1^ (dry weight)					Citrate wt%	
	Cl^−^	NO_3_^−^	PO_4_^3−^	SO_4_^2−^	Sum anions wt%		
1	1295	700	711	4167	0.69	81.3	
2	1280	863	1178	903	0.42	79.2	
3	1097	369	593	599	0.27	68.9	
	**Cations** **μg g**^**−1**^ **(dry weight)**						
**Sample**	**K**^**+**^	**Mg**^**2+**^	**Ni**^**2+**^	**Ca**^**2+**^	**Co**^**2+**^	**Sum cations wt%**	**Total balance wt%**
1	649	821	145560	1300	1869	15.1	96.4
2	1211	810	168514	1871	1449	17.4	96.6
3	1532	595	154944	1960	557	16.1	85.0

Results obtained with ICP-AES, High Performance Liquid Chromatography (HPLC) and ion chromatography.
