# Supplementary material for: Nickel biopathways in tropical nickel hyperaccumulating trees from Sabah (Malaysia)
Source: Sci Rep. 2017 Feb 16;7:41861. doi: 10.1038/srep41861 (PMC5311975; doi:10.1038/srep41861)
Supplement: Supplementary Information [file srep41861-s1.pdf]

Nickel biopathways in tropical nickel hyperaccumulating trees from Sabah (Malaysia)

Antony van der Ent[1,2]*, Damien L. Callahan[3], Barry N. Noller[1], Jolanta Mesjasz-Przybylowicz[4], Wojciech J. Przybylowicz[4,5], Alban Barnabas[4], Hugh H. Harris[6*]

[1]Centre for Mined Land Rehabilitation, Sustainable Minerals Institute, The University of Queensland, Queensland, Australia

[2]Université de Lorraine–INRA, Laboratoire Sols et Environnement, UMR 1120, France

[3]Deakin University, Geelong, Australia. School of Life and Environmental Sciences, Centre for Chemistry and Biotechnology (Burwood Campus), Victoria, Australia

[4]Materials Research Department, iThemba LABS, National Research Foundation, Somerset West, South Africa

[5]AGH University of Science and Technology, Faculty of Physics & Applied Computer Science, Krakow, Poland

[6]Department of Chemistry, The University of Adelaide, South Australia, Australia

* a.vanderent@uq.edu.au & hugh.harris@adelaide.edu.au

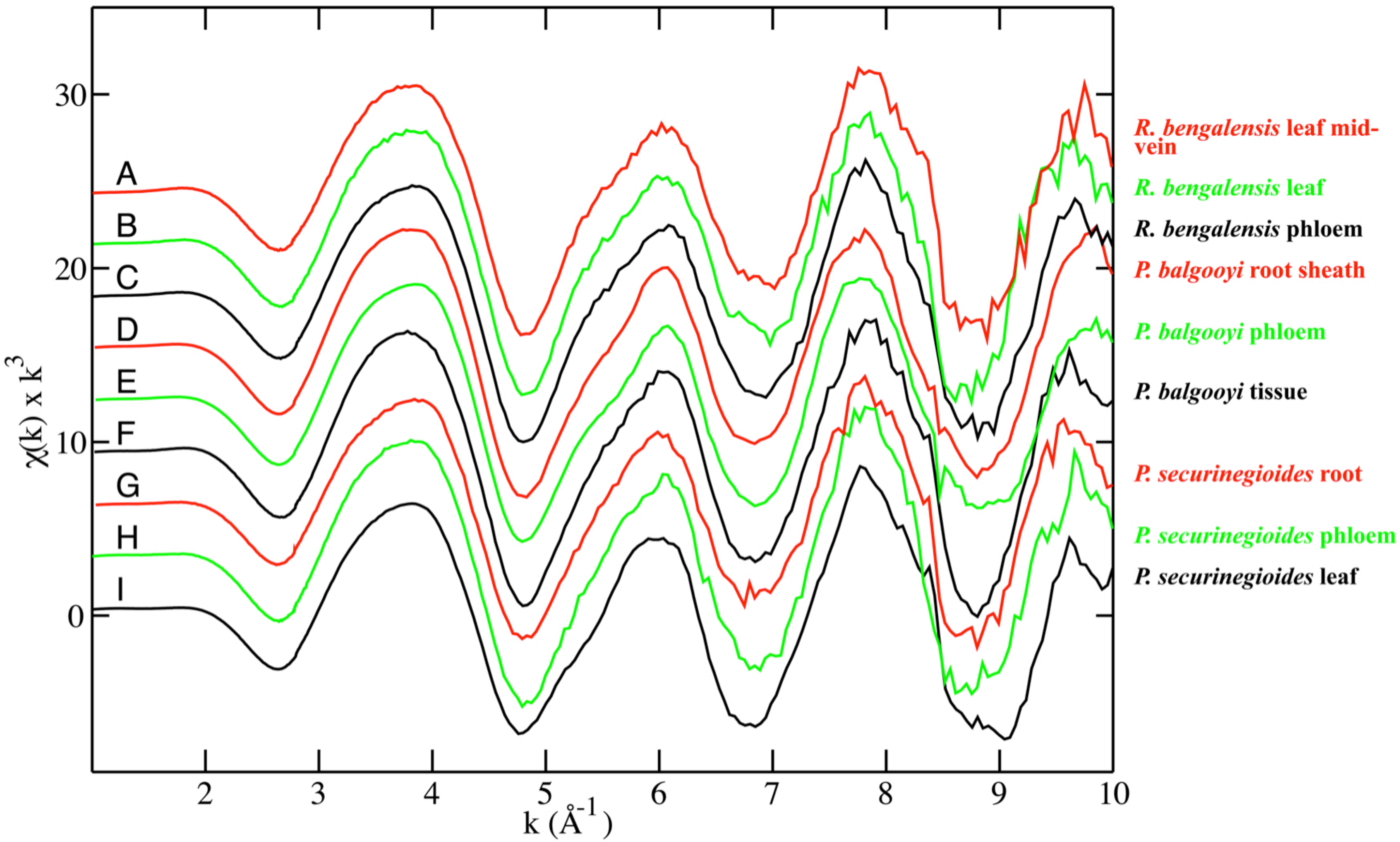

Supplementary Figure 1. Ni K-edge extended X-ray absorption structure for **A**, *Rinorea bengalensis* leaf mid-vein tissue; **B**, *Rinorea bengalensis* leaf tissue; **C**, *Rinorea bengalensis* phloem tissue; **D**, *Phyllanthus balgooyi* root sheath tissue; **E,** *Phyllanthus balgooyi* phloem tissue; **F**, *Phyllanthus balgooyi* leaf tissue; **G**, *Phyllanthus securinegioides* root tissue; **H**, *Phyllanthus securinegioides* phloem tissue; **I**, *Phyllanthus securinegioides* leaf tissue.

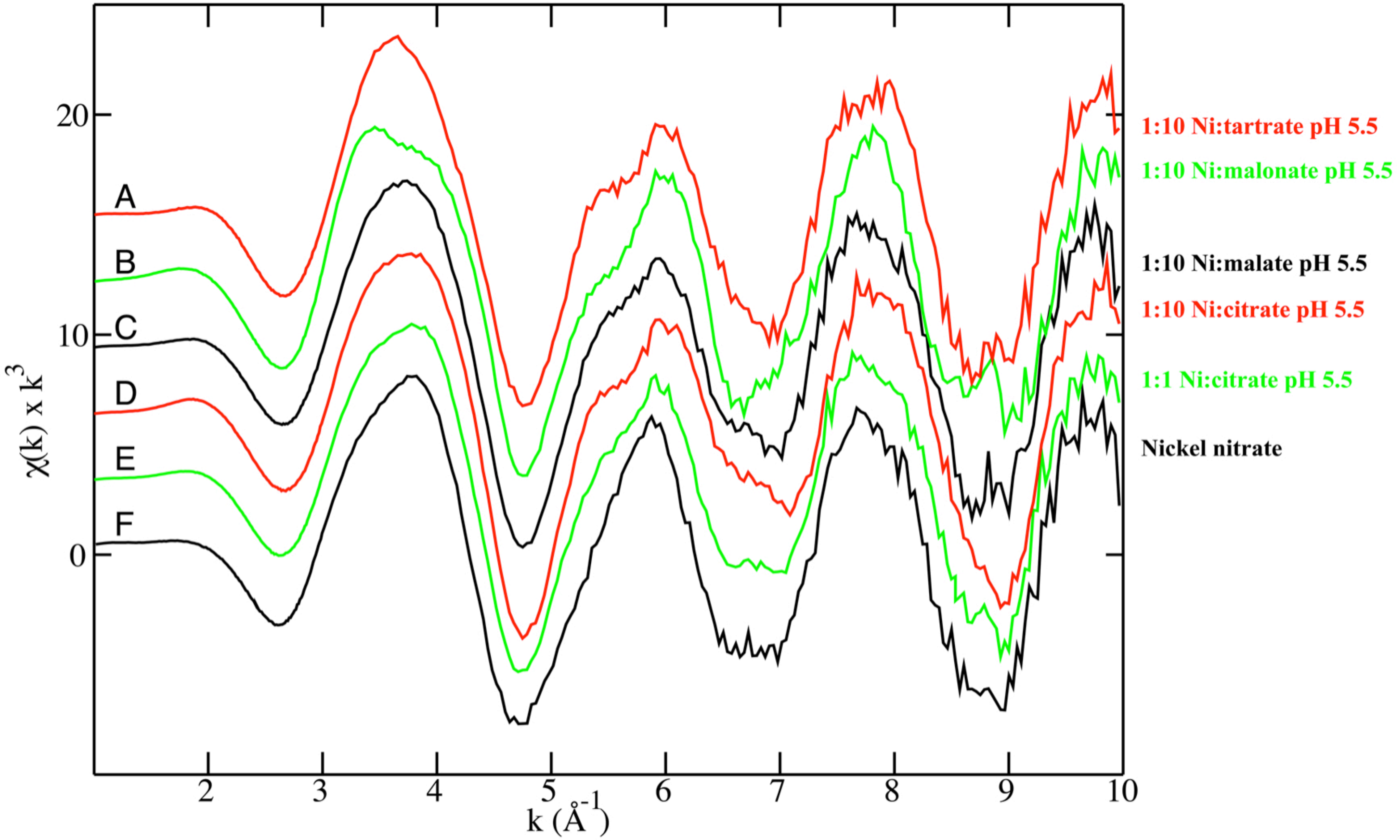

Supplementary Figure 2. Ni K-edge extended X-ray absorption structure for **A**, 1:10 Ni:tartrate in aqueous solution at pH 5.5; **B**, 1:10 Ni:malonate in aqueous solution at pH 5.5; **C**, 1:10 Ni:malate in aqueous solution at pH 5.5; **D**, 1:10 Ni:citrate in aqueous solution at pH 5.5; **E**, 1:1 Ni:citrate in aqueous solution at pH 5.5; **F**, Ni:nitrate in aqueous solution (*i.e.* $[Ni(H_2O)_6]^{2+}$.

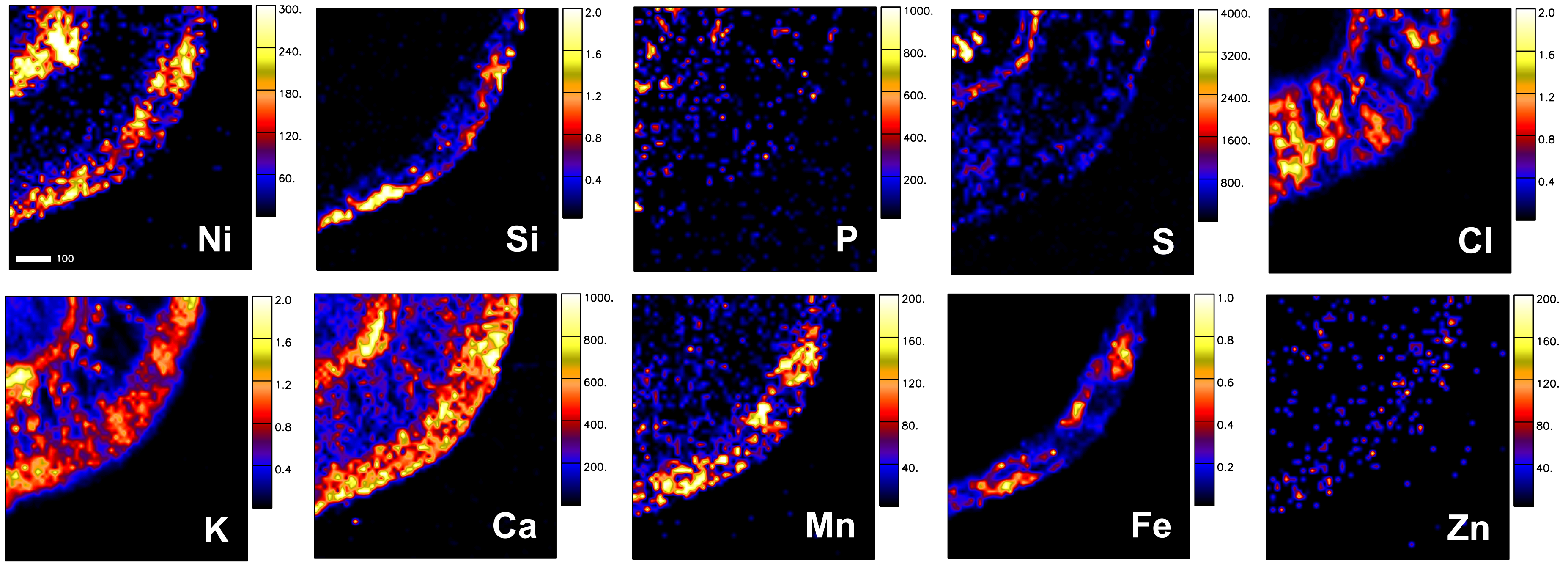

Supplementary Figure 3. Elemental maps of root cross-section of *Phyllanthus balgooyi.* Concentration scale in wt% dry weight (for Si, Cl, K and Fe) and µg g^{-1} dry weight (for Ni, P, S, Ca, Mn and Zn). Scale bar – 100 µm.

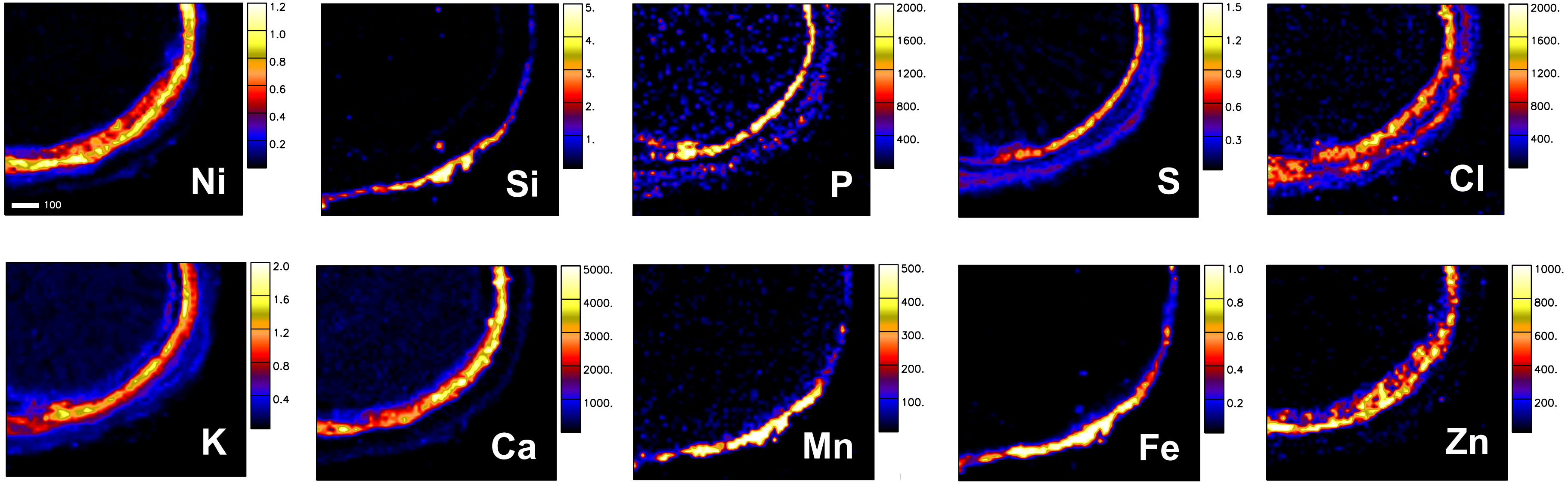

Supplementary Figure 4. Elemental maps of root cross-section of *Phyllanthus securinegioides.* Concentration scale in wt% dry weight (for Ni, Si, S, K and Fe) and μg g^{-1} dry weight (for P, Cl, Ca, Mn and Zn). Scale bar – 100 μm.

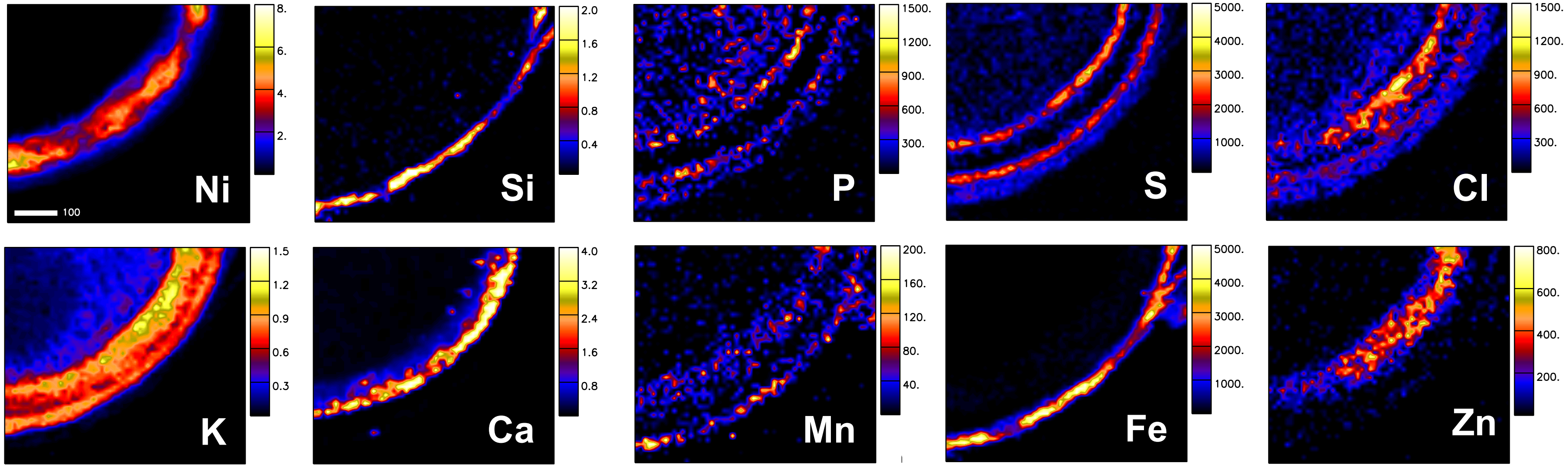

Supplementary Figure 5. Elemental maps of root cross-section of *Rinorea bengalensis.* Concentration scale in wt% dry weight (for Ni, Si, K and Ca) and µg g^{-1} dry weight (for P, S, Cl, Mn, Fe and Zn). Scale bar – 100 µm.

Phyllanthus balgooyi (stem section)

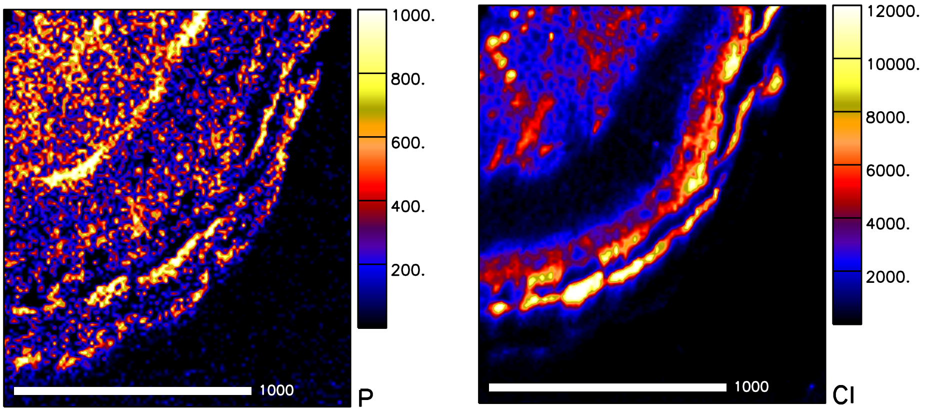

Phyllanthus securinegoides (stem section)

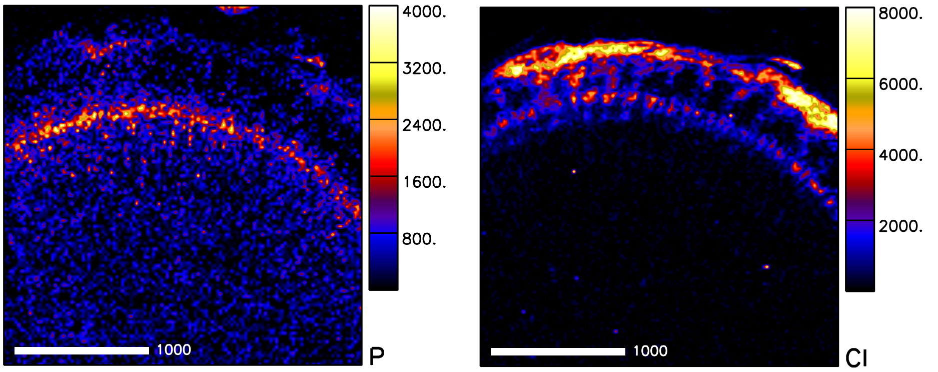

Rinorea bengalensis (stem section)

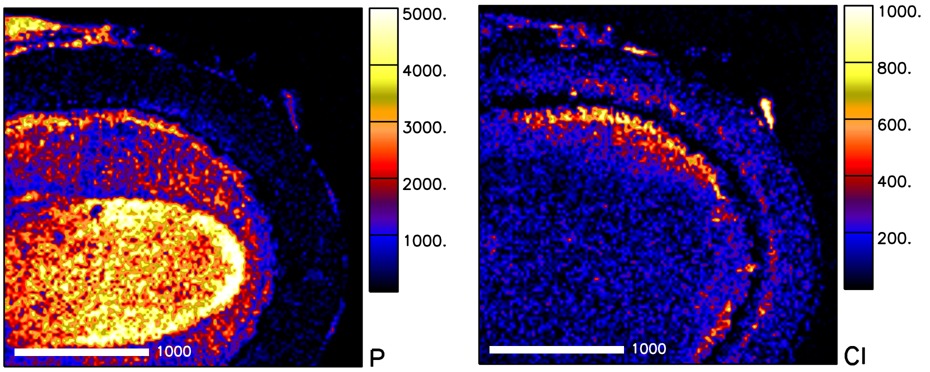

Supplementary Figure 6. Elemental maps of stem cross-sections of *Phyllanthus balgooyi, Phyllanthus securinegioides* and *Rinorea bengalensis.* Concentration scale in µg g^{-1} dry weight. Scale bar – 1000 µm.

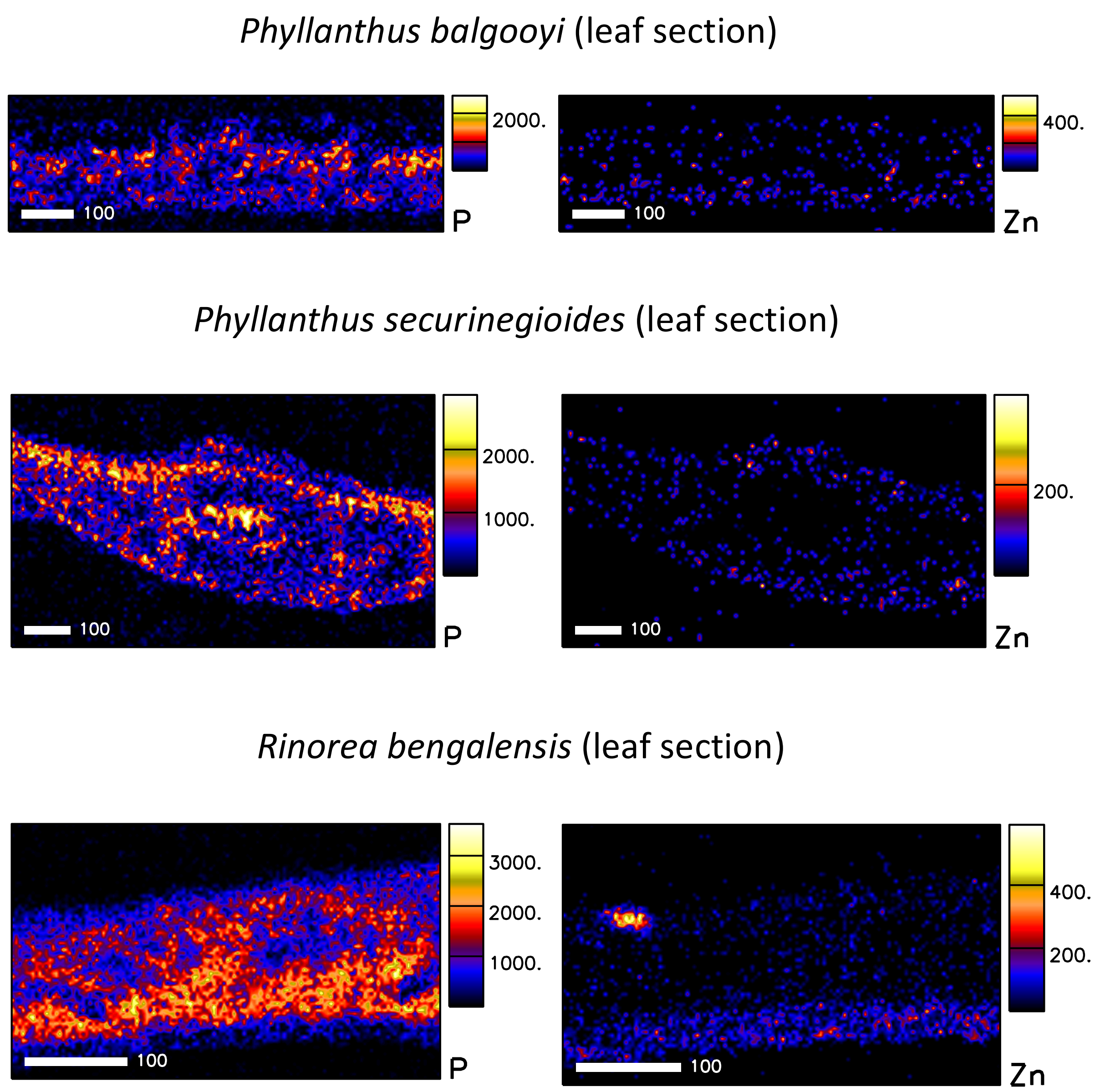

Supplementary Figure 7. Elemental maps of leaf cross-sections of *Phyllanthus balgooyi, Phyllanthus securinegioides* and *Rinorea bengalensis.* Concentration scale in µg g^{-1} dry weight. Scale bar – 100 µm.
